# Pathogen profiles and co-detection characteristics of acute respiratory infections based on active surveillance in a large district of southern China

**DOI:** 10.3389/fpubh.2026.1854385

**Published:** 2026-07-10

**Authors:** Yixiong Chen, Sheng Zhang, Zhifeng Ma, Ziqi Wang, Jinfeng Liu, Dandan Niu, Jingjing Li, Xindong Zhang, Bing Chen, Meng Ren

**Affiliations:** 1 Bao’an Center for Disease Control and Prevention, Shenzhen, China; 2 Bao’an Center for Public Health Service, Shenzhen, China

**Keywords:** active surveillance, acute respiratory infections, co-detection patterns, co-detection rates, pathogen profiles

## Abstract

**Objective:**

The objective of this study is to determine the multipathogen profiles and co-detection characteristics among patients with acute respiratory infections (ARIs) in a large district of southern China.

**Methods:**

This multicenter study conducted active surveillance of patients with acute respiratory infections (ARIs) at eight sentinel hospitals in a large district of southern China from August 1, 2024, to December 31, 2025. The study employed multiplex polymerase chain reaction (PCR) technology to screen a series of respiratory specimens for 20 respiratory pathogens. Multi-stage logistic regression models were constructed to assess co-detection associations between pathogens at the individual level.

**Results:**

The median [interquartile range (IQR)] age of the 6,199 patients included in the study was 24 [5, 33] years. The detection rate and co-detection rate for any pathogen were 62.5% (3,876/6,199) and 18.5% (1,148/6,199), respectively. The highest viral detection rate (69.1%, 993/1,437) was observed in children under five, and the highest bacterial detection rate (47.6%, 487/1,022) was observed in school-age children. The three most prevalent viral pathogens were identified as rhinovirus (RV), influenza A virus (IAV) and severe acute respiratory syndrome-coronavirus 2 (SARS-CoV-2). The three most prevalent bacterial pathogens were identified as *Haemophilus influenzae* (*H. influenzae*), *Streptococcus pneumoniae* (SPN) and Group A Streptococcus (GAS). The three most prevalent co-detection pairs were SPN with *H. influenzae* (3.4%, 210/6,199), RV with *H. influenzae* (2.8%, 171/6,199), and RV with SPN (2.0%, 125/6,199 cases). The detection rates for RV, SARS-CoV-2 and GAS in outpatients were higher than those in inpatients, while the detection rates for RSV, enterovirus (EV), bocavirus (HBoV) and SPN in inpatients were higher than those in outpatients. Virus–virus pairs, such as IAV combined with RV (OR = 0.17, 95%CI: 0.11–0.25) and with RSV (OR = 0.06, 95% CI: 0.02–0.27) exhibited negative co-detection associations. Furthermore, bacteria-bacteria pairs showed positive co-detection associations, such as the combination of SPN and *H. influenzae* (OR = 2.12, 95% CI: 1.74–2.57).

**Conclusion:**

The characteristics of the pathogens vary depending on the patient’s age, the nature of the pneumonia, and the type of medical care sought. Virus-virus pairs showed negative co-detection associations, whereas bacteria-bacteria pairs showed positive co-detection associations.

## 1 Introduction

Acute respiratory infection (ARI) is defined as a clinical acute illness characterized by an abrupt onset of any respiratory symptoms caused by various pathogens. The primary symptoms encompass a range of respiratory ailments, including but not limited to: cough, expectoration, dyspnea, pharyngitis, and rhinorrhea (1). ARI constitutes a significant global public health concern, marked by elevated incidence and mortality, thereby representing a persistent and substantial threat to human health. According to statistics from the World Health Organization, ARIs are the fourth leading cause of death worldwide and the second leading cause in low-income countries. A systematic analysis based on the 2021 Global Burden of Disease Study indicates that, globally, there were an estimated 344 million episodes of lower respiratory tract infections in 2021, resulting in approximately 2.18 million deaths (2). In recent decades, various novel respiratory pathogens have emerged worldwide (3, 4), frequently triggering infectious disease outbreaks, while the epidemiological patterns of these pathogens continue to evolve. This further underscores the critical importance of long-term, continuous pathogen surveillance for ARI in the prevention and control of respiratory infectious diseases.

The epidemiological distribution of respiratory pathogens is influenced by multiple factors, and their epidemiological characteristics exhibit variability across different periods and regions. Regional surveillance studies have confirmed these dynamic patterns (5). For instance, influenza A viruses (IAV) manifest distinct seasonal patterns in northern China, typically peaking during the cold season (October through January). Conversely, in southern China, the epidemic curve exhibits two peaks, one in January and the other in August, respectively (5). In the preceding decade, a considerable number of studies have documented the various pathogens responsible for ARIs (6–24). However, the composition of these pathogen profiles exhibits substantial variations across different geographical regions, attributable to various factors including geography, season, and population composition. Particularly following the COVID-19 pandemic, the epidemiological characteristics of respiratory pathogens remain unclear. There is presently a lack of systematic analysis of recent multi-pathogen surveillance data.

Furthermore, extant surveillance systems are characterized by certain limitations with regard to coverage and timeliness. For example, China’s surveillance system prioritizes specific pathogens, including but limited to influenza, measles, mumps, pertussis, tuberculosis, and scarlet fever. Due to the relatively fixed nature of the diseases under surveillance and the limited range of pathogens encompassed, it is challenging to provide a timely and comprehensive reflection of the complex changes in the respiratory pathogen spectrum. Consequently, the establishment of a multi-pathogen surveillance system for ARIs has become an urgent necessity in the field of public health.

A district in southern China, located in the heart of the Guangdong-Hong Kong-Macao Greater Bay Area, has a permanent population of 4.47 million, equivalent in size to a medium-sized prefecture-level city. The region’s high population density and high mobility provide a typical epidemiological research setting for the transmission and surveillance of ARI. The district has established an active surveillance system for ARI based on multipathogen testing. This study aims to clarify the epidemiological characteristics of pathogen detection and co-detection associations among ARI patients in the region. It will provide a foundation for refining the ARI surveillance system and optimizing prevention and control strategies.

## 2 Materials and methods

### 2.1 Patient recruitment

Between August 1, 2024 and December 31, 2025, we conducted active surveillance for ARIs across eight secondary or tertiary public hospitals in a district of southern China. Active surveillance means our team proactively planned sampling and coordinated with hospitals rather than relying on routine reports. The eight sentinel hospitals are distributed throughout the district, ensuring good representativeness. The study enrolled outpatients and inpatients with a preliminary diagnosis of ARI and/or pneumonia at fever clinics, respiratory departments, emergency departments, or intensive care units, following the 2016 clinical practice guidelines by the Chinese Thoracic Society (25). Patients with non-infectious respiratory diseases such as asthma or cancer were excluded.

### 2.2 Specimen collection and testing

Respiratory specimens including oropharyngeal swabs, nasopharyngeal swabs and bronchoalveolar lavage fluid were collected from patients within 72 h of presentation and prior to treatment (26). Oropharyngeal and nasopharyngeal swabs were prioritized for patients with upper respiratory tract infections, whereas bronchoalveolar lavage fluid was prioritized for those with lower respiratory tract infections. To ensure specimen balance and representativeness, we used systematic sampling for upper respiratory tract infections, collecting two specimens from each hospital every Tuesday, Thursday, and Saturday, specifically the first and third patients by order of arrival. For pneumonia cases, we used quota sampling, collecting a fixed number of specimens from each hospital each month. On designated sampling days, trained physicians followed standardized protocols to enroll eligible patients consecutively. Specimen collection spanned all 12 months of the year and involved hospitals covering every street of Bao’an district, ensuring reasonable representativeness in terms of both seasonal and geographical distribution. A questionnaire was used to collect information on department visited, patient demographics, clinical symptoms, specimen type and informed consent.

All specimens were processed in a Biosafety Level 2 laboratory by trained staff following standard operating procedures and quality control protocols. Qualitative detection of 20 respiratory pathogens was performed using a multiplex fluorescent PCR kit (Shenzhen United Medical Technology Co., Ltd.) on the ABI QuantStudio 5 system. Specifically, the panel comprised 14 viruses [severe acute respiratory syndrome-coronavirus 2 (SARS-CoV-2), influenza A viruses (IAV), influenza B viruses (IBV), respiratory syncytial virus (RSV), adenovirus (AdV), human metapneumovirus (HMPV), parainfluenza virus (PIV), human coronavirus NL63 (HCoV-NL63), human coronavirus HKU1 (HCoV-HKU1), human coronavirus 229E (HCoV-229E), human coronavirus OC43 (HCoV-OC43), human bocavirus (HBoV), rhinovirus (RV), and enterovirus (EV)], four bacteria [group A streptococcus (GAS), *bordetella pertussis* (*B. pertussis*), *streptococcus pneumoniae* (SPN), and *haemophilus influenzae* (*H. influenzae*)] as well as *chlamydia pneumoniae* (*C. pneumoniae*) and mycoplasma pneumoniae (MP). A detailed list of the target genes is provided in Supplementary Table S1. Positive and negative controls were included in each run. Results were recorded in real time and verified by two independent technicians. A sample was considered positive if it showed a typical amplification curve with a Ct value ≤ 38, provided that the controls were valid; otherwise, it was considered negative. This method has been validated, with a limit of detection of 200 copies/mL (viruses, mycoplasmas, and chlamydiae) or 1 × 10^3^ CFU/mL (bacteria).

### 2.3 Statistical analysis

The study first employed descriptive analysis. Qualitative variables were presented as frequencies and percentages, while quantitative variables were described as the mean ± standard deviation for normally distributed data or the median and interquartile range for skewed data, depending on the data distribution. The subjects of the study were stratified by age into the following groups: children (<5 years), school-aged children (5–17 years), adults (18–59 years), and older adults (≥60 years) for analysis. To compare differences in pathogen detection rates across groups, the chi-square test was used, adjusted with Bonferroni correction. Additionally, Join-point regression analysis (JPR) was employed to explore trends in pathogen detection rates across age groups.

The present study employed a multi-stage logistic regression method to assess co-detection associations between pathogens at the individual host level (5, 6). In the initial stage, each pathogen Y was designated as the dependent variable, while another non-Y pathogen X was utilized as the independent variable. The following were included as covariates: age (qualitative variable), sex, disease severity (presence or absence of pneumonia), seasonal characteristics (month of the year, qualitative variable) and specimen types (oropharyngeal swabs, nasopharyngeal swabs and bronchoalveolar lavage fluid). In the absence of adjustment for the presence of multiple pathogens, this preliminary analysis identified 190 potential pairwise co-detection patterns among 20 pathogens. In the second stage, the test results for all remaining non-Y pathogens were treated as explanatory variables simultaneously, while adjusting for the aforementioned covariates, in an effort to eliminate the confounding effects of the simultaneous presence of multiple pathogens. The interaction between two pathogens was confirmed only when both A and B were significant in models where they served as independent and dependent variables, respectively. In the third stage, the results from the first two stages were synthesized to form a final conclusion, and the nature of the interaction was determined using odds ratios (OR). Due to the cross-sectional nature of our PCR data, all analyses describe statistical patterns of pathogen co-detection. Therefore, we interpret ORs greater than 1 as positive co-detection associations and ORs less than 1 as negative co-detection associations.

## 3 Results

### 3.1 Participants

**Figure 1:**
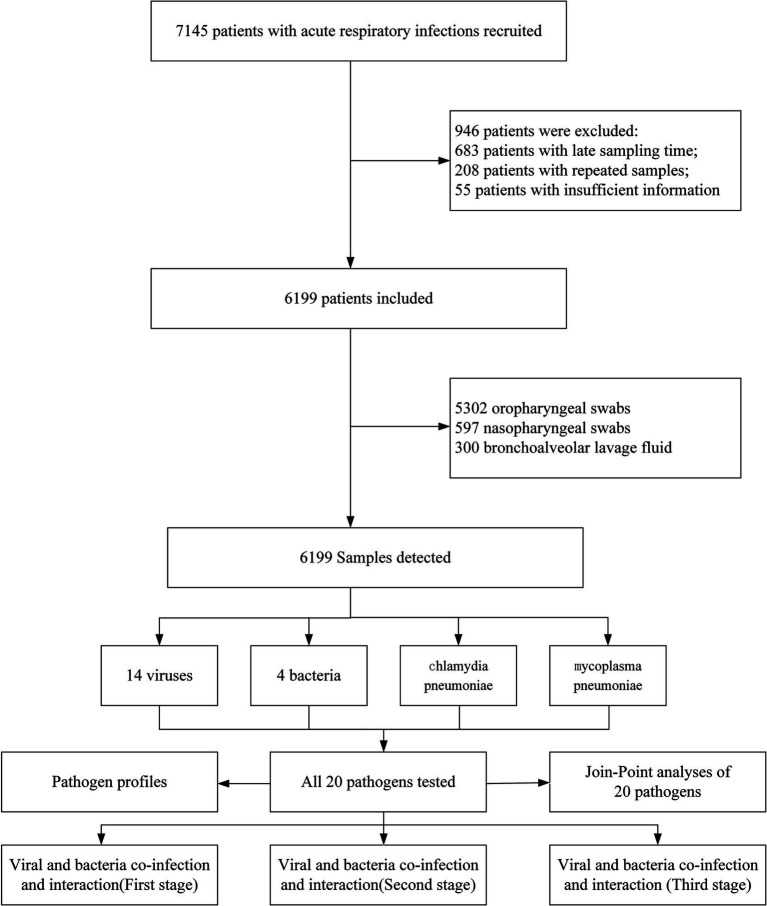
Flowchart of the data collection and analysis procedures.

A total of 7,145 patients with ARI were recruited from eight sentinel hospitals in Bao’an District, Shenzhen. Of these, 946 patients were excluded due to late or duplicate specimen collection or insufficient information. Ultimately, 6,199 patients were included in the study (Figure 1). The median age of the patients was 24 (5–33) years, and 54.1% (3,353/6,199) were male. Among them, 15.1% (938/6,199) were hospitalized, and 9.9% (614/6,199) were diagnosed with pneumonia.

### 3.2 Pathogen detection rate

The detection rate for any virus among patients with ARI was 48.6% (3,011/6,199). Significant differences were observed across age groups (*χ*^2^ = 419.172, *p* < 0.001), and the detection rate showed a decreasing trend with increasing age (Cochran-Armitage trend test: *Z* = −20.466, *p* < 0.001). Pairwise comparisons revealed that the viral detection rates were higher among children under 5 years of age (69.1%, 993/1437), school-aged children (54.5%, 557/1022), and adults (40.3%, 1,395/3,465) compared to older adults (24.0%, 66/275). The study revealed no statistically significant differences in the viral detection rates between outpatients and inpatients (48.4%, 2,545/5,261; 49.7%, 466/938; *χ*^2^ = 0.492, *p* = 0.483), or between males and females (48.3%, 1,618/3,353; 49.0%, 1,393/2,846; *χ*^2^ = 0.267, *p* = 0.605) (Table 1, Supplementary Table S2).

**Table 1:** Pathogen detection rates among patients with acute respiratory infections in Bao’an district, Shenzhen, 2024–2025.

Groups	Any virus detected			Any bacteria detected			Any pathogens detected			Two or more pathogens detected		
	All (*N* = 6,199)	Pneumonia (*N* = 614)	Non pneumonia (*N* = 5,585)	All (*N* = 6,199)	Pneumonia (*N* = 614)	Non pneumonia (*N* = 5,585)	All (*N* = 6,199)	Pneumonia (*N* = 614)	Non pneumonia (*N* = 5,585)	All (*N* = 6,199)	Pneumonia (*N* = 614)	Non pneumonia (*N* = 5,585)
All (*N* = 6,199)	3,011 (48.6)	265 (43.2)	2,746 (49.2)	1754 (28.3)	161 (26.2)	1,593 (28.5)	3,876 (62.5)	340 (55.4)	3,536 (63.3)	1,148 (18.5)	106 (17.3)	1,042 (18.7)
Sex^#^												
Male (*N* = 3,353)	1,618 (48.3)	148 (41.9)	1,470 (49.0)	957 (28.5)	90 (25.5)	867 (28.9)	2080 (62.0)	189 (53.5)	1891 (63.0)	646 (19.3)	65 (18.4)	581 (19.4)
Female (*N* = 2,846)	1,393 (49.0)	117 (44.8)	1,276 (49.4)	797 (28.0)	71 (27.2)	726 (28.1)	1796 (63.1)	151 (57.8)	1,645 (63.6)	502 (17.6)	41 (15.7)	461 (17.8)
Age group												
Children (<5 years) (*N* = 1,437)	993 (69.1)	175 (74.8)	818 (68.0)	539 (37.5)	86 (36.8)	453 (37.7)	1,162 (80.9)	199 (85.0)	963 (80.0)	501 (34.9)	77 (32.9)	424 (35.2)
School age children (5–17 years) (*N* = 1,022)	557 (54.5)	36 (53.7)	521 (54.6)	487 (47.6)	33 (49.2)	454 (47.5)	793 (77.6)	54 (80.6)	739 (77.4)	327 (32)	18 (26.9)	309 (32.4)
Adult (18–59 years) (*N* = 3,465)	1,395 (40.3)	36 (17.5)	1,359 (41.7)	685 (19.8)	26 (12.6)	659 (20.2)	1824 (52.6)	58 (28.2)	1766 (54.2)	305 (8.8)	5 (2.4)	300 (9.2)
Old people (≥60 years) (*N* = 275)	66 (24.0)	18 (16.8)	48 (28.6)	43 (15.6)	16 (15.0)	27 (16.1)	97 (35.3)	29 (27.1)	68 (40.5)	15 (5.45)	6 (5.6)	9 (5.4)
Case type												
Outpatients (*N* = 5,261)	2,545 (48.4)	45 (34.4)	2,500 (48.7)	1,422 (27.0)	17 (13.0)	1,405 (27.4)	3,272 (62.2)	55 (42.0)	3,217 (62.7)	910 (17.3)	9 (6.9)	901 (17.6)
Inpatients (*N* = 938)	466 (49.7)	220 (45.6)	246 (54.1)	332 (35.4)	144 (29.8)	188 (41.3)	604 (64.4)	285 (59.0)	319 (70.1)	238 (25.4)	97 (20.1)	141 (31.0)

The data were presented as the number (%) unless otherwise indicated. The percentages may not total 100 because of rounding.

The detection rate for any bacterial pathogen among patients with ARI was 28.3% (1,754/6,199). There were significant differences between age groups (*χ*^2^ = 394.732, *p* < 0.001), but no clear trend in detection rates with age (Cochran-Armitage trend test: *Z* = 0.899, *p* = 0.369). Paired comparisons revealed that the bacterial detection rates were higher in children under 5 years of age (37.5%, 539/1,437) and school-aged children (47.6%, 487/1,022) than in older adults group (15.6%, 43/275). The positive rate of any bacterial pathogen in hospitalized patients (35.4%, 332/938) was higher than that in outpatients (27.0%, 1,422/5,261) (*χ*^2^ = 27.047, *p* < 0.001) (Table 1, Supplementary Table S2).

The detection rate for any viral or bacterial pathogen among patients with ARI was 62.5% (3,876/6,199). A statistically significant discrepancy was observed among the different age groups (*χ*^2^ = 536.913, *p* < 0.001). Furthermore, the detection rate exhibited a declining trend with increasing age (Cochran-Armitage trend test: *Z* = −7.7487, *p* < 0.001). Pairwise comparisons revealed that the detection rate for any viral or bacterial pathogen was higher among children under 5 years of age (80.9%, 1,162/1,437), school-aged children (77.6%, 793/1,022), and adults (52.6%, 1,824/3,465) than among older adults (35.3%, 97/275). The detection rates for viral and bacterial pathogens exhibited no significant difference between outpatients and inpatients (62.2%, 3,272/5,261; 64.4%, 604/938; *χ*^2^ = 1.550, *p* = 0.213) (Table 1, Supplementary Table S2).

**Figure 2:**
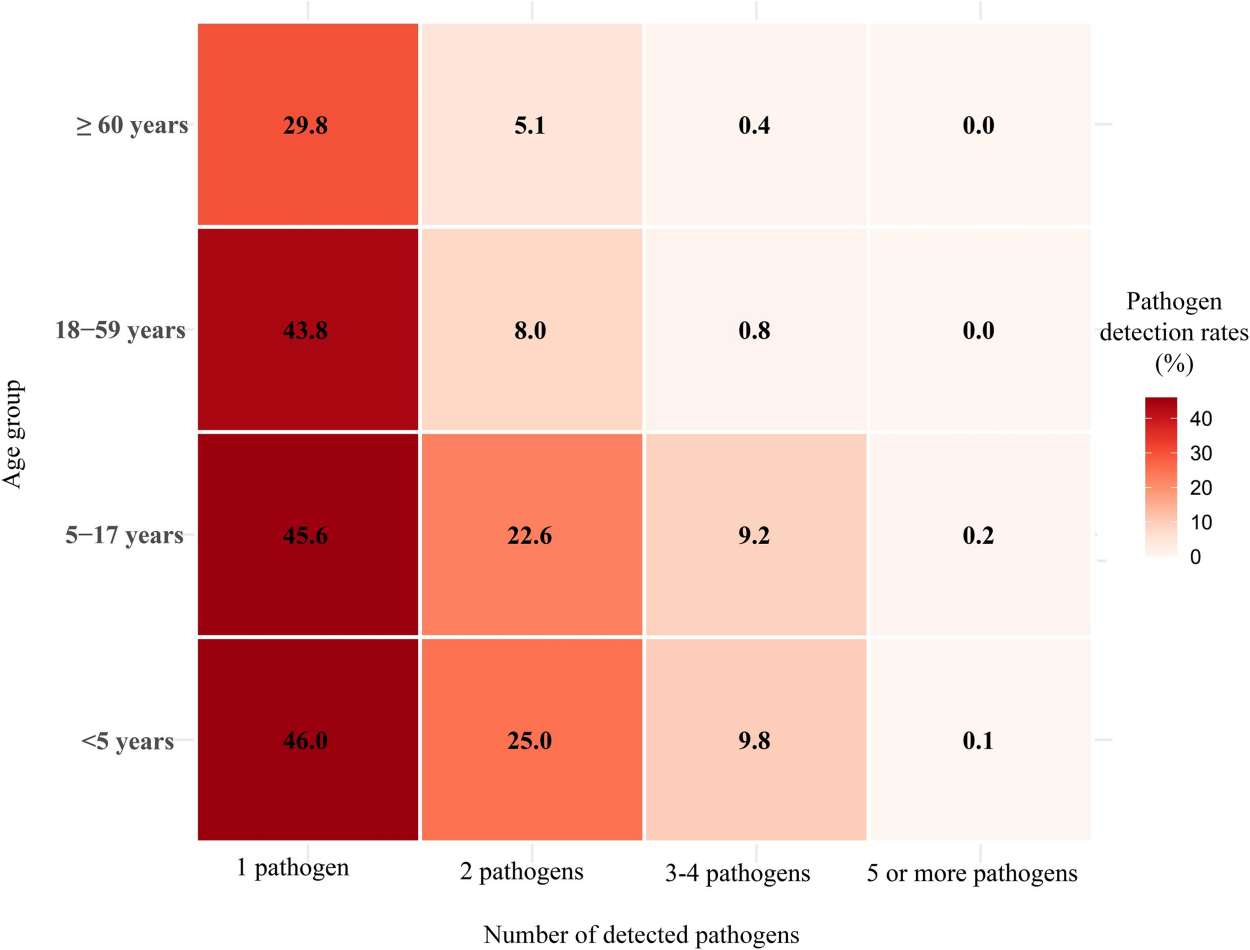
Distribution of single and multiple pathogen detection rates across age groups in Bao’an district, Shenzhen, 2024–2025.

The co-detection rate for two or more respiratory pathogens was 18.5% (1,148/6,199) (Table 1). A statistically significant discrepancy was observed among the various age groups (*χ*^2^ = 625.350, *p* < 0.001) (Supplementary Table S2). Furthermore, the co-detection rate exhibited a declining trend with increasing age (Cochran-Armitage trend test: *Z* = −9.592, *p* < 0.001). Pairwise comparisons revealed that the co-detection rates for two or more respiratory pathogens were higher in children under 5 years of age (34.9%, 501/1,437) and school-aged children (32.0%, 327/1,022) than in older adults group (5.5%, 15/275). The co-detection rate for two, three to four, and five or more pathogens were 14.2% (881/6,199), 4.2% (263/6,199), and 0.06% (4/6,199), respectively. Statistically significant differences were identified in the distribution of pathogen counts across different age groups (*χ*^2^ = 612.723, *p* < 0.05). Among patients under 5 years of age, the detection rates for a single, two, three to four, and five or more pathogens were 46.0% (661/1,437), 25.0% (359/1,437), 9.8% (141/1,437), and 0.1% (1/1,437), respectively (Figure 2).

### 3.3 Pathogen profiles

Supplementary Table S3 shows the detection rates of 20 respiratory pathogens among patients with acute respiratory infections. From the perspective of pathogen characteristics, the detection rate for pathogens associated with Class B notifiable infectious diseases was 8.4% (520/6,199), including two pathogens: SARS-CoV-2 and *B. pertussis*, with detection rates of 8.36% (518/6,199) and 0.03% (2/6,199), respectively. Furthermore, three pathogens associated with Class C notifiable infectious diseases were detected, including IAV, IBV, and EV, with detection rates of 11.7% (727/6,199), 1.1% (66/6,199), and 1.7% (108/6,199), respectively.

**Figure 3:**
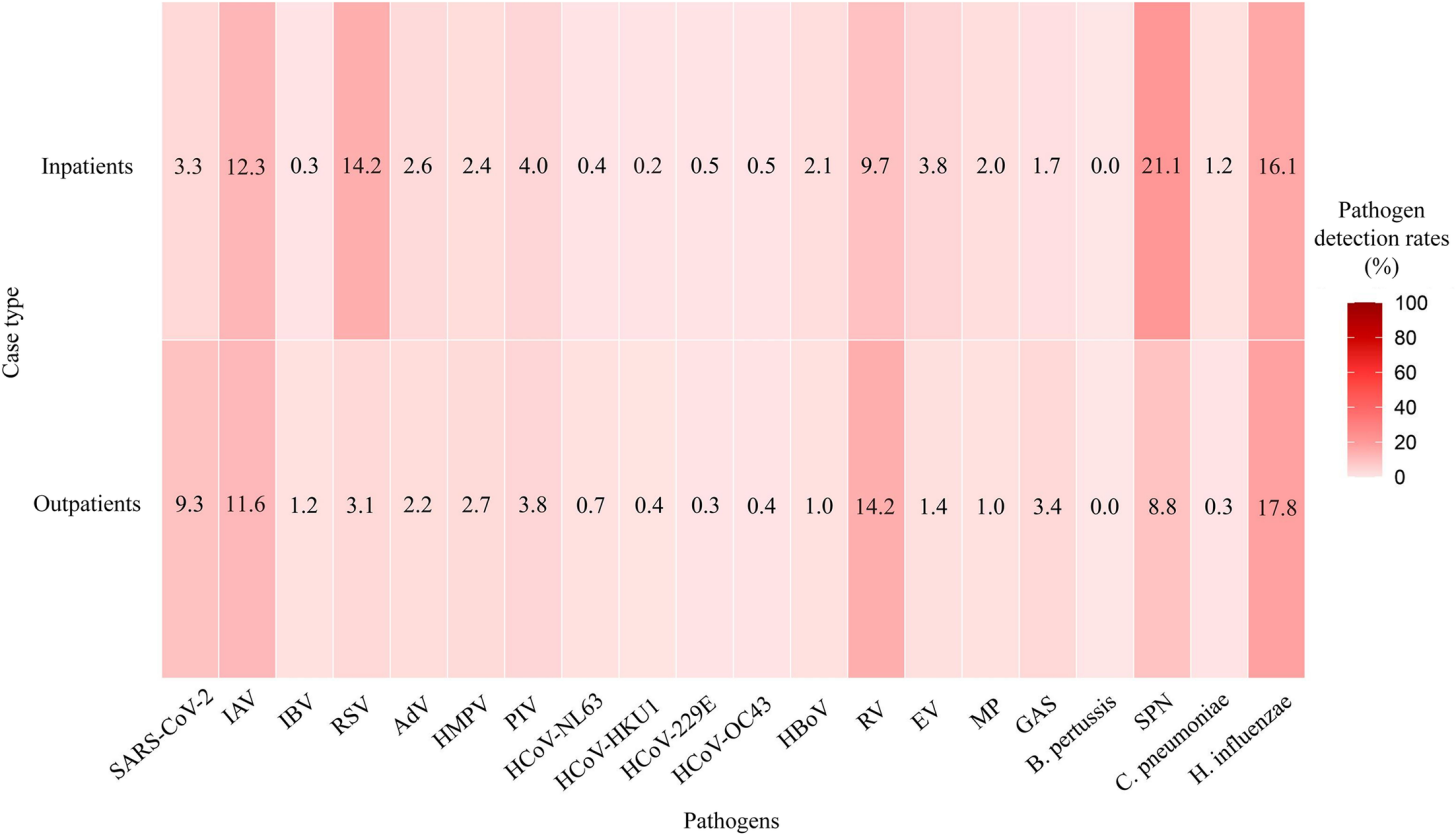
Distribution of pathogen detection rates among inpatients and outpatients in Bao’an district, Shenzhen, 2024–2025.

Test results for 20 key respiratory pathogens showed that RV, IAV and SARS-CoV-2 had the highest detection rates, at 13.6% (840/6,199), 11.7% (727/6,199), and 8.4% (518/6,199), respectively (Supplementary Table S3). The top three viruses detected among outpatients were RV (14.2%, 749/5,261), IAV (11.6%, 612/5,261), and SARS-CoV-2 (9.3%, 487/5,261), respectively. Among inpatients, the top three viruses were RSV (14.2%, 133/938), IAV (12.3%, 115/938), and RV (9.7%, 91/938), respectively. The detection rates for both RV (*χ*^2^ = 11.30, *p* = 0.001) and SARS-CoV-2 (*χ*^2^ = 36.70, *p* < 0.001) were found to be higher among outpatients. Higher detection rates were observed for RSV (*χ*^2^ = 120.43, *p* < 0.001), enterovirus (EV) (*χ*^2^ = 14.53, *p* < 0.001), and HBoV (*χ*^2^ = 4.10, *p* = 0.043) among inpatients (Supplementary Table S4, Figure 3).

**Figure 4:**
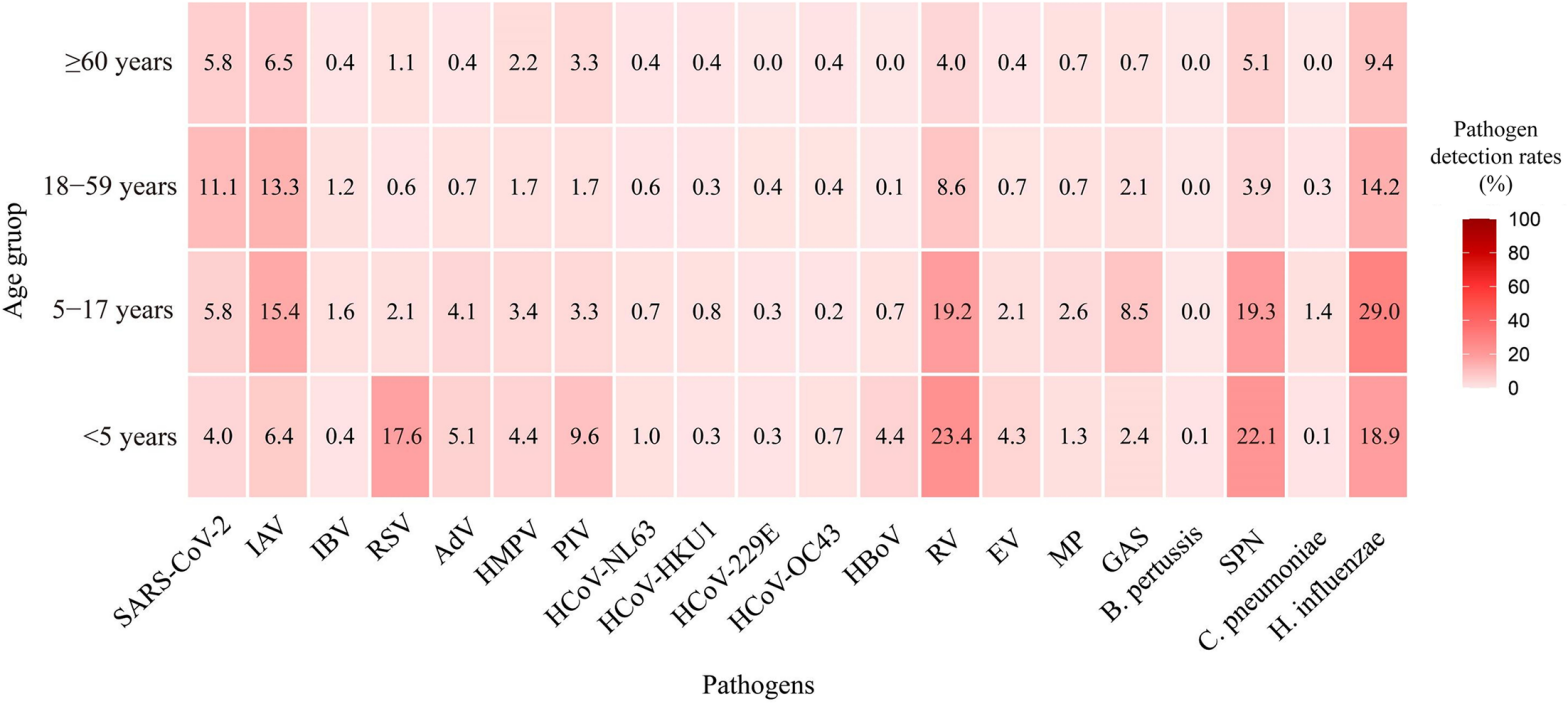
Pathogen detection rates by age in Bao’an district, Shenzhen, 2024–2025.

Among children under 5 years of age, the top four viruses were RV (23.4%, 336/1437), followed by RSV (17.6%, 253/1,437), PIV (9.6%, 138/1,437), and IAV (6.4%, 92/1437). The top four viruses by detection rate among school-aged children were RV (19.2%, 196/1,022), IAV (15.4%, 157/1,022), SARS-CoV-2 (5.8%, 59/1022), and AdV (4.1%, 42/1022). The top three viruses by detection rate among adults and older adult were consistent: IAV, SARS-CoV-2, and RV. The detection rates among adults were 13.3% (460/3465), 11.1% (386/3465), and 8.6% (297/3465), while the detection rates among older adult were 6.5% (18/275), 5.8% (16/275), and 4.0% (11/275), respectively (Figure 4, Supplementary Table S5).

**Figure 5:**
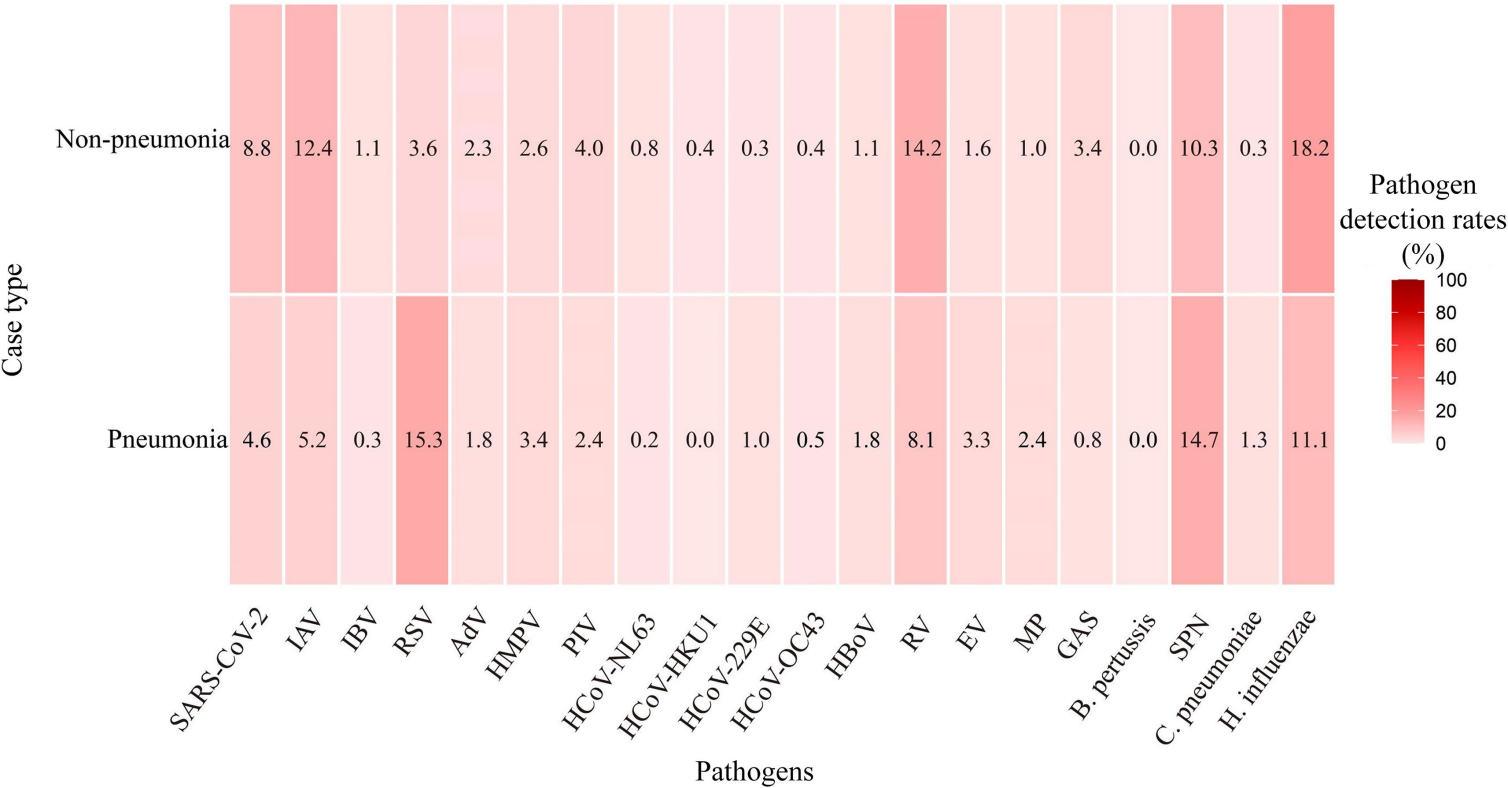
Pathogen detection rates by pneumonia status in Bao’an district, Shenzhen, 2024–2025.

Among patients with pneumonia and those without pneumonia, the three viruses with the highest detection rates were RSV (15.3%, 94/614), RV (8.1%, 50/614), and IAV (5.2%, 32/614); RV (14.2%, 790/5585), IAV (12.4%, 695/5585), and SARS-CoV-2 (8.8%, 490/5585), respectively (Figure 5, Supplementary Table S6). RSV (*χ*^2^ = 142.32, *p* < 0.001), EV (*χ*^2^ = 7.60, *p* = 0.006), and HCoV-229E (*χ*^2^ = 5.30, *p* = 0.021) were detected at higher rates in patients with pneumonia. In contrast, non-pneumonia patients exhibited higher detection rates for RV (*χ*^2^ = 16.60, *p* < 0.001), IAV (*χ*^2^ = 27.30, *p* < 0.001), and SARS-CoV-2 (*χ*^2^ = 12.70, *p* < 0.001) (Supplementary Table S6).

**Figure 6:**
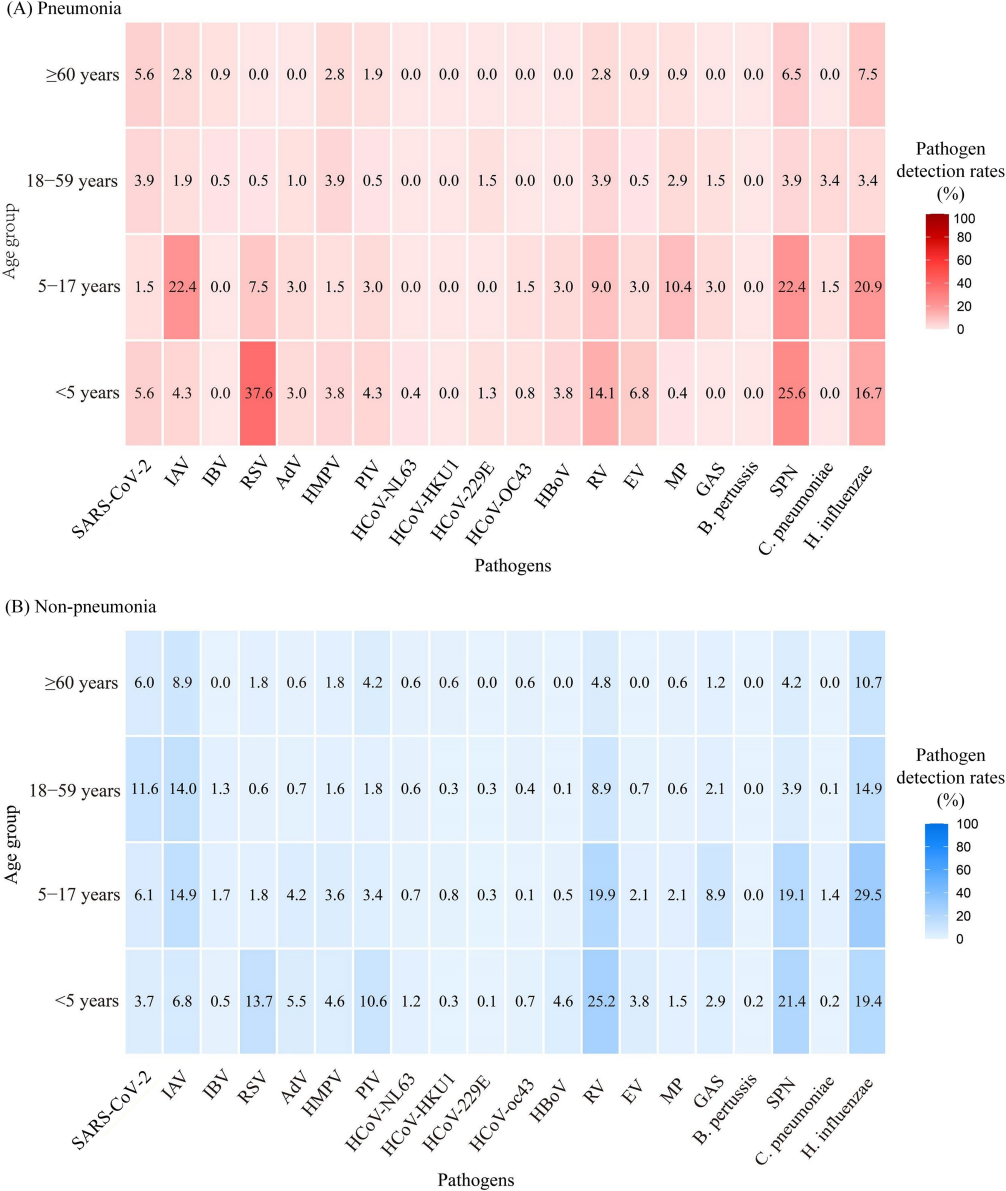
Pathogen detection rates by age and pneumonia status in Bao’an district, Shenzhen, 2024–2025.

Among children under 5 years of age with pneumonia, the two most prevalent viruses were RSV (37.6%, 88/234) and RV (14.1%, 33/234). The RV detection rate among children with pneumonia (14.1%, 33/234) was significantly lower than that among children without pneumonia (25.2%, 303/1203) (*χ*^2^ = 10.46, 0.001 < *p* < 0.01). Among school-aged children with pneumonia, the most prevalent viruses were IAV (22.4%, 15/67) and RV (9.0%, 6/67). The RV detection rate in the pneumonia group (9.0%, 6/67) was significantly lower than that observed in children without pneumonia (19.9%, 190/955) (*χ*^2^ = 4.76, 0.01 < *p* < 0.05). The three most prevalent viruses identified in adult patients with pneumonia were SARS-CoV-2, HMPV, and RV, each with a detection rate of 3.9% (8/206). Among older adult patients, the four most common viruses were SARS-CoV-2 (5.6%, 6/107), IAV (2.8%, 3/107), HMPV (2.8%, 3/107), and RV (2.8%, 3/107) (Figure 6, Supplementary Table S7).

The three bacteria with the highest detection rates were *H. influenzae* (17.5%, 1,086/6,199), SPN (10.7%, 663/6,199), and GAS (3.2%, 196/6,199) (Supplementary Table S3). The most prevalent bacterial species among outpatients were *H. influenzae* (17.8%, 935/5261), SPN (8.8%, 465/5261), and GAS (3.4%, 180/5261). The most prevalent bacterial pathogens among inpatients were SPN (21.1%, 198/938), *H. influenzae* (16.1%, 151/938), and GAS (1.7%, 16/938). The detection rate for GAS was higher among outpatients (*χ*^2^ = 5.70, *p* = 0.017). The detection rate for SPN was higher among inpatients (*χ*^2^ = 83.55, *p* < 0.001) (Figure 3, Supplementary Table S4).

Among children under 5 years of age, the common bacterial species were consistent, but the order differed. SPN (22.1%, 318/1,437) ranked first, followed by *H. influenzae* (18.9%, 272/1,437) and GAS (2.4%, 35/1,437). The top three bacteria by detection rate were consistent across school-aged children, adults, and older adult: *H. influenzae*, SPN, and GAS. The detection rates for school-aged children were 29.0% (296/1,022), 19.3% (197/1,022), and 8.5% (87/1,022), respectively. For adults, the rates were 14.2% (492/3,465), 3.9% (134/3,465), and 2.1% (72/3,465), respectively. For older adults, the rates were 9.4% (26/275), 5.1% (14/275), and 0.7% (2/275), respectively. The detection rates for *H. influenzae* (*χ*^2^ = 152.73, *p* < 0.001), SPN (*χ*^2^ = 412.35, *p* < 0.001), and GAS (*χ*^2^ = 121.03, *p* < 0.001) all demonstrated significant differences across age groups. Among these, the detection rate for SPN demonstrated a gradual decline with increasing age (Cochran-Armitage trend test: *Z* = −18.93, *p* < 0.001). *H. influenzae* (Cochran-Armitage trend test: *Z* = −4.28, *p* < 0.001) and GAS (Cochran-Armitage trend test: *Z* = −3.56, *p* < 0.001) were associated with age but did not follow a single linear trend. The maximum detection rates for both were observed in the school-aged population (Figure 4, Supplementary Table S5).

The top three pathogens by detection rate were consistent between pneumonia and non-pneumonia patients. For pneumonia patients, the most prevalent bacterial pathogens were SPN (14.7%, 90/614), *H. influenzae* (11.1%, 68/614), and GAS (0.8%, 5/614). For non-pneumonia patients, they were *H. influenzae* (18.2%, 1,018/5,585), SPN (10.3%, 573/5,585), and GAS (3.4%, 191/5,585) (Figure 5, Supplementary Table S6). The SPN detection rate was significantly higher in the pneumonia group (*χ*^2^ = 11.74, *p* < 0.01). However, the detection rates for *H. influenzae* (*χ*^2^ = 18.05 *p* < 0.001) and GAS (*χ*^2^ = 11.80, *p* < 0.01) were higher among patients without pneumonia (Supplementary Table S6).

Among children under 5 years of age, the top two bacteria in both the pneumonia and non-pneumonia groups were SPN and *H. influenzae*, with detection rates of 25.6% (60/234) and 16.7% (39/234) for pneumonia patients, and 21.4% (258/1203) and 19.4% (233/1203) for non-pneumonia patients, respectively. There was no statistically significant difference in the detection rates of these two pathogens between the two groups (*p* > 0.05). Among school-aged children, the two predominant bacterial species were consistent for both pneumonia and non-pneumonia patients. For pneumonia, they were SPN (22.4%, 15/67) and *H. influenzae* (20.9%, 14/67). In contrast, among non-pneumonia cases, *H. influenzae* (29.5%, 282/955) emerged as the predominant bacterial pathogen, followed by SPN (19.1%, 182/955). Moreover, a statistically significant discrepancy in the detection rates of these two pathogens between the two groups was not observed (*p* > 0.05). Among adults, the top three pathogens in pneumonia patients were SPN (3.9%, 8/206), *C. pneumoniae* (3.4%, 7/206), and *H. influenzae* (3.4%, 7/206). The most prevalent bacterial species in non-pneumonia patients were *H. influenzae* (14.9%, 485/3,259), SPN (3.9%, 126/3,259), and GAS (2.1%, 69/3,259). A comparison of the detection rates for SPN between the two groups revealed no statistically significant difference (*p* > 0.05). However, the detection rate for *H. influenzae* was significantly higher in the non-pneumonia group (*χ*^2^ = 12.88, *p* < 0.001). Among older adults, the predominant bacterial pathogens identified in both pneumonia and non-pneumonia patients were *H. influenzae* and SPN. The positive rates for pneumonia patients were 7.5% (8/107) and 6.5% (7/107), respectively, while those for non-pneumonia patients were 10.7% (18/168) and 4.2% (7/168), respectively. A statistically significant difference in the detection rates for these two pathogens between the two groups was not observed (*p* > 0.05) (Figure 6, Supplementary Table S7).

### 3.4 Pathogen co-detection rates and pairwise associations

**Figure 7:**
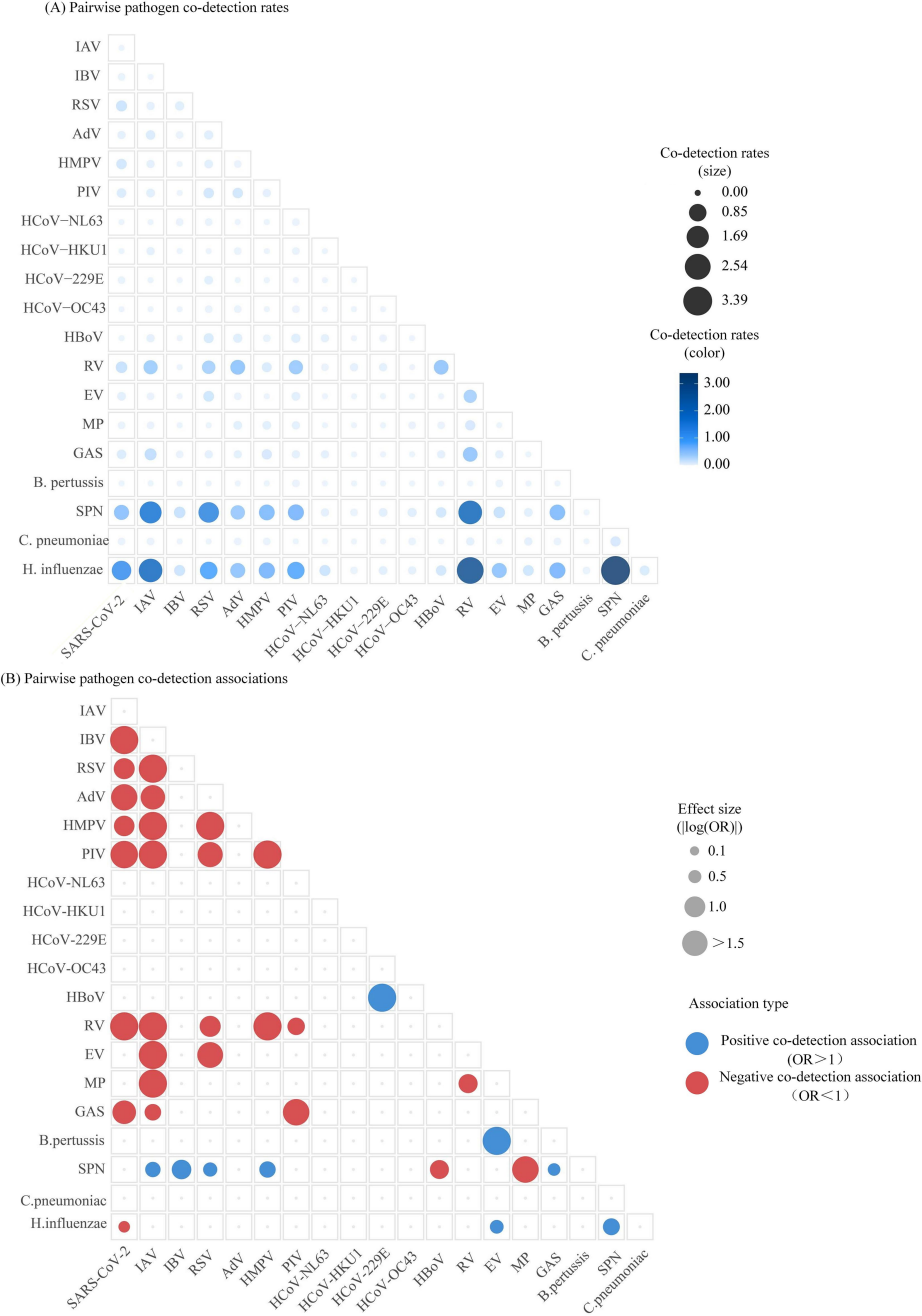
Pathogen co-detection rates and associations among patients with acute respiratory infections in Bao’an district, Shenzhen, 2024–2025. **(A)** Pairwise pathogen co-detection rates; **(B)** pairwise pathogen co-detection associations.

The three most prevalent pathogens involved in co-detections were SPN and *H. influenzae* (3.4%, 210/6,199), RV and *H. influenzae* (2.8%, 171/6,199), and RV and SPN (2.0%, 125/6,199 cases) (Figure 7A, Supplementary Table S8). Viral-viral pairs primarily exhibited a negative co-detection association, such as between IAV and RV (OR = 0.17, 95% CI: 0.11–0.25, *p* < 0.001), IAV and RSV (OR = 0.06, 95% CI: 0.02–0.27, *p* < 0.001), SARS-CoV-2 and RSV (OR = 0.27, 95% CI: 0.13–0.54, *p* < 0.001), and SARS-CoV-2 and AdV (OR = 0.18, 95% CI: 0.04–0.76, *p* < 0.001). Only HCoV-229E and HBoV (OR = 15.05, 95% CI: 1.46–155.39, *p* < 0.05) exhibited positive co-detection associations. The predominant pattern among bacterial pairs was positive co-detection. For instance, the presence of SPN was associated with a higher likelihood of GAS (OR = 1.74, 95% CI: 1.16–2.61, *p* < 0.05), as well as with *H. influenzae* (OR = 2.12, 95% CI: 1.74–2.57, *p* < 0.001). Virus-bacteria pairs can show either positive or negative co-detection associations. For example, HMPV and SPN (OR = 2.28, 95% CI: 1.49–3.48, *p* < 0.05), RSV and SPN (OR = 1.93, 95% CI: 1.40–2.67, *p* < 0.001) exhibited a positive co-detection association, while IAV and GAS (OR = 0.37, 95% CI: 0.21–0.66, *p* < 0.05), HBoV and SPN (OR = 0.44, 95% CI: 0.21–0.96, *p* < 0.05) exhibited a negative co-detection association (Figure 7B, Table 2, Supplementary Tables S2, S8). The results of co-detection associations from the oropharyngeal swab subgroup were largely consistent with the above (Supplementary Table S9).

**Table 2:** Pairwise pathogen co-detection associations among patients with acute respiratory infections in Bao’an district, Shenzhen, 2024–2025, adjusted for multiple pathogens.

Pathogens	SARS-CoV-2	IAV	IBV	RSV	AdV	HMPV	PIV	HCoV-NL63	HCoV-HKU1	HCoV-229E
SARS-CoV-2	—	0	0.077^#&^	0.266^#&^	0.179^#&^	0.253^#&^	0.160^#&^	0	0	0.348
IAV	0	—	0	0.063^#&^	0.214^#&^	0.084^#&^	0.087^#&^	0	0.353	0
IBV	0.075^#&^	0	—	1.505	0.648	0	0	0.810	0	0
RSV	0.288^#&^	0.076^#&^	1.063	—	0.272	0.104^#&^	0.174^#&^	0.104	0	1.093
AdV	0.186^#&^	0.202^#&^	0.594	0.287	—	0.160	0.640	0	1.322	0
HMPV	0.247^#&^	0.079^#&^	0	0.145^#&^	0.139	—	0.117^#&^	0	0	0
PIV	0.145^#&^	0.083^#&^	0	0.181^#&^	0.590	0.117^#&^	—	0.287	0.834	0
HCoV-NL63	0	0	0.866	0.111	0	0	0.373	—	0	0
HCoV-HKU1	0	0.317	0	0	1.206	0	0.823	0	—	0
HCoV-229E	0.428	0	0	1.353	0	0	0	0	0	—
HCoV-OC43	0	0	0	0.644	0.931	0	0.672	0	0	0
HBoV	0	0.322	0	0.737	0.839	0.875	0.588	2.233	0	6.851
RV	0.146^#&^	0.170^#&^	0	0.357^#&^	0.769	0.128^#&^	0.329^#&^	0.123	0.296	1.021
EV	0.330	0.090^#&^	0	0.239^#&^	0.243	0	0.309	0	0	0
*H. influenzae*	0.777	0.890	0.619	0.733	1.103	1.533^*^	1.077	1.213	0.228	0.913
GAS	0.223^#&^	0.374^#&^	0.296	0.180^#^	0.262	0.760	0.171^#&^	1.306	0	0
*B. pertussis*	0	0	0	0	0	0	11.017	0	0	0
SPN	1.004	2.143^*&^	3.587^*&^	1.763^*&^	1.294	2.425^*&^	1.153	0.460	0.324	1.953
MP	0.267	0.083^#&^	0	0	0.904	2.639	0.398	0	0	0
*C. pneumoniae*	0.941	0.230	0	0	1.074	0.909	0	0	0	0

Pathogens	HCoV-OC43	HBoV	RV	EV	*H. influenzae*	GAS	*B. pertussis*	SPN	MP	*C. pneumoniae*
SARS-CoV-2	0	0	0.154^#&^	0.355	0.715^#^	0.229 ^#&^	0	1.061	0.316	0.946
IAV	0	0.306	0.166^#&^	0.086^#&^	0.920	0.369 ^#&^	0	2.100 ^*&^	0.071 ^#&^	0.144
IBV	0	0	0	0	0.643	0.278	0	3.110 ^*&^	0	0
RSV	0.904	0.697	0.254 ^#&^	0.209 ^#&^	0.874	0.178	0	1.930 ^*&^	0	0
AdV	0.734	0.841	0.818	0.255	1.060	0.255	0	1.299	0.760	0.997
HMPV	0	0.741	0.112 ^#&^	0	1.413	0.690	0	2.278 ^*&^	2.114	0.965
PIV	0.564	0.563	0.325 ^#&^	0.261	1.071	0.175 ^#&^	6.070	1.249	0.373	0
HCoV-NL63	0	3.188	0.137	0	1.238	1.782	0	0.366	0	0
HCoV-HKU1	0	0	0.318	0	0.226	0	0	0.406	0	0
HCoV-229E	0	15.045^*^	1.038	0	0.845	0	0	1.655	0	0
HCoV-OC43	-	0	0	0	0.410	0	0	1.615	1.297	8.770
HBoV	0	-	1.596	0	0.537	0.877	0	0.444 ^#&^	0.489	0
RV	0	1.566	-	1.219	1.022	0.713	0	1.184	0.289 ^#&^	0.248
EV	0	0	1.201	-	1.676 ^*&^	0.445	29.465 ^*&^	0.605	0	0
*H. influenzae*	0.366	0.537	1.017	1.619^*&^	-	0.782	12.261	2.105 ^*&^	0.563	1.099
GAS	0	1.014	0.676	0.477	0.823	-	0	1.686 ^*&^	0	0.654
*B. pertussis*	0	0	0	62.578^*&^	3.592	0	-	0	0	0
SPN	1.489	0.418 ^#&^	1.162	0.530	2.116 ^*&^	1.742 ^*&^	0	-	0.266 ^#&^	2.652
MP	1.900	0.483	0.330 ^#&^	0	0.602	0	0	0.282 ^#&^	-	0.914
*C. pneumoniae*	9.191	0	0.252	0	1.209	0.529	0	2.691^*^	1.848	-

This table lists only the odds ratios for the pathogens.^*^positive co-detection associations with two-sided *p* < 0.05; ^#^negative co-detection associations with *p* < 0.05. Column names represented the dependent variables, and row names represented the independent variables. ^&^represented the pairwise co-detection associations between ‘X’ and ‘Y’, as both the dependent and independent variables were consistent. A statistically significant co-detection association was referred to as a “multipathogen-adjusted association”.

### 3.5 Trends in pathogen detection rates by age

Among the predominant viruses, the detection rates of both IAV and SARS-CoV-2 showed a trend of initially increasing and then decreasing with age, with cutoff ages of 7 years (APC_1_ = 1.94, APC_2_ = −0.10) and 26 years (APC_1_ = 0.35, APC_2_ = −0.13), respectively. In contrast, the RV detection rate among patients under 27 years of age decreased with increasing age (APC = −0.58), while the rate of decline slowed among patients aged 27 and older (APC = -0.12). Among patients under 6 years of age, the RSV detection rate decreased with increasing age (APC = −4.07), while the rate of decline slowed among patients aged 6 and older (APC = −0.01) (Supplementary Figure S1).

Among the predominant bacteria, the detection rates for *H. influenzae* and GAS demonstrated a trend of first increasing and then decreasing with age, with cutoff ages of 4 years (APC_1_ = 3.17, APC_2_ = −0.34) and 6 years (APC_1_ = 1.02, APC_2_ = −0.14), respectively. In contrast, the SPN detection rate exhibited a negative association with age in patients under 23 years old (APC = −0.96) and a positive association with age in patients aged 23 years or older (APC = 0.07) (Supplementary Figure S2).

## 4 Discussion

This study analyzed the recent pathogen profiles and co-detection patterns among patients with ARI in a large district of southern China. The overall detection rate for any respiratory pathogen was 62.5%, and the co-detection rate was 18.5%. RV, IAV, and SARS-CoV-2 were the most commonly detected viruses, while *H. influenzae*, SPN, and GAS were the most frequently detected bacteria. The profiles of pathogens exhibited variation across age groups and between patients with and without pneumonia. Negative co-detection associations predominated among virus-virus pairs, whereas positive co-detection associations predominated among bacteria-bacteria pairs. However, both types of associations were observed among virus-bacteria pairs.

Multiplex PCR is a screening tool employed for the prevention and control of ARI, primarily utilized for the rapid detection of common pathogens. This study, employing multiplex PCR technology, found that the overall detection rate for any pathogen among patients with ARIs was 62.5%, and the co-detection rate with two or more pathogens was 18.5%. However, the 37.5% negative rate observed may be related to sampling quality, low pathogen load, or pathogens not covered by the 20-target panel. To address these limitations, the utilization of higher-throughput technologies for identification in the future is a potential solution (27, 28). As a high-throughput, precise screening method that covers the core pathogen spectrum and drug resistance genes, tNGS can effectively address the detection blind spots of multiplex PCR. Meanwhile, mNGS, as a comprehensive and unbiased ultimate validation technique, is particularly suitable for identifying difficult-to-diagnose, critical, or emerging pathogens.

This study found that RV, IAV, and SARS-CoV-2 were the top three viruses in terms of detection rates, consistent with results from regions such as Beijing, Zhejiang, and Hubei in China (6–24). This pathogen profile may be associated with the seasonal epidemiological patterns of common respiratory viruses, the population’s immune background, and the combined effects of viral mutations and control measures on the transmission intensity of different pathogens (29–31). Our findings indicated that RV exhibited the highest detection rate. RV infection is characterized primarily by mild upper respiratory symptoms and is the leading cause of the common cold, accounting for one-third to one-half of all cold cases. It is prone to causing cluster outbreaks in enclosed settings such as daycare centers and schools (32, 33). Furthermore, patients with mild RV infections have a high rate of seeking medical care and a large baseline population, often resulting in high detection rates in community-based active surveillance (34). Besides, the antigenic characteristics of RV are subject to constant mutation, giving rise to new variants on a frequent basis. This phenomenon can result in the recurrent occurrence of infections, with various strains potentially circulating concurrently within the population. This observation might offer a rationale for the elevated detection rate observed among individuals experiencing ARI. IAV demonstrated the second-highest detection rate. It is evident that IAV is susceptible to antigenic drift, a process that facilitates its evasion of the human immune system and expedites its dissemination among susceptible populations, resulting in seasonal peaks. Consequently, during periods of high influenza incidence, the vaccination of priority groups and the initiation of early antiviral treatment should be reinforced (35). The SARS-CoV-2 ranked third in detection rate. This phenomenon may be related to the establishment of a mixed immune barrier in the population, resulting from widespread vaccination and prior natural infection. This has led to a substantial reduction in the transmission intensity and disease severity of SARS-CoV-2 (36). The virus has now become analogous to common respiratory viruses, exhibiting a pattern of routine circulation. Our findings suggest that ARI prevention and control should shift from single-pathogen to multi-pathogen surveillance (5). Further studies on the disease burden are warranted to inform targeted prevention strategies.

The present study found that *H. influenzae*, SPN, and GAS had the highest detection rates, consistent with findings from Shenzhen, Jiangsu, and Shandong in China (12, 37–39). However, the presence of these bacteria should not be automatically interpreted as etiological infection, as colonization is common. They are opportunistic pathogens that can cause invasive disease when immunity is compromised, the airway mucosal barrier is damaged, or viral co-infections occurs (40). The high detection rate for *H. influenzae* likely reflects its high colonization prevalence in children and adolescents (41), as well as its transmission in crowded settings (40). Following airway epithelial damage, its adhesion and colonization in the lower respiratory tract may increase (42), but detection does not necessarily imply invasive infection. SPN is a recognized cause of pneumonia and other ARIs (43). It is estimated that this pathogen caused 1.18 million deaths worldwide in 2016. Its pathogenicity is influenced by capsular serotype, antibiotic resistance, and population immunity levels. During the peak epidemic season in winter and spring, co-infection with respiratory viruses may exacerbate disease severity. However, given that SPN is also a common colonizing bacterium of the upper respiratory tract, the fact that it ranked second in detection rate in this study via PCR does not necessarily imply the presence of invasive infection. GAS ranked third in detection. It causes pharyngitis, tonsillitis, and scarlet fever, with high transmissibility. Though severe disease is rare, non-purulent complications such as acute glomerulonephritis and rheumatic fever remain a concern (44). As with other bacteria detected in this study, PCR positivity alone should not be interpreted as active infection. Future work should address bacterial disease burden and antimicrobial use to guide multiple-pathogens control strategies.

The pathogen spectrum varies across different age groups. The highest viral detection rate was observed in children under 5 years of age, while school-aged children have the highest bacterial detection rate. RSV is more prevalent among children under 5 years of age, whereas IAV predominates among school-aged children, adults, and older adults. This pattern may be related to the epidemiological characteristics of different viruses and the immune status of the population. It is widely accepted that children under 5 years of age possess an underdeveloped immune system and delicate respiratory mucosal barriers, thus rendering them the primary susceptible population for RSV (10, 23). RSV has been observed to cause lower respiratory tract infections including bronchiolitis and pneumonia, with a notable age-specific clustering pattern. IAV are highly transmissible and prone to antigenic drift, leading to widespread transmission among school-aged children, adults, and older adults in schools, households, and public settings. Among these groups, older adults face a higher risk of severe illness following infection due to declining immune function and the presence of underlying medical conditions (45). These age-related differences suggest that the prevention and control of ARI should implement targeted intervention strategies tailored to specific populations and pathogens, thereby reducing the overall disease burden.

Complex co-detection patterns exist among respiratory pathogens. Consistent with previous findings, viruses tended to be negatively co-detected with each other (6), possibly related to limited cell surface receptors and interferon responses that create an exclusionary barrier (46). In contrast, consistent with the finding from previous study (6), bacteria-bacteria pairs primarily showed positive co-detection, which may reflect biofilm formation and quorum sensing-mediated metabolic complementation and collective defense (47). Virus-bacteria pairs were even more complex, showing either positive or negative co-detection. This variation may be associated with factors such as the type of pathogen, the sequence of infection, the host immune status, and changes in the airway microenvironment (48). While these findings provide useful regional surveillance data and identify statistical co-detection patterns, they do not establish true biological interactions or causality among pathogens. Further studies incorporating longitudinal data, pathogen load dynamics, infection timing, and mechanistic experiments are needed to elucidate the underlying biological mechanisms and to inform integrated prevention and treatment strategies (48).

This study was the first to establish a multi-site monitoring network at the county level, and employed multi-pathogen detection techniques to conduct tests on dozens of respiratory pathogens. Multiplex pathogen detection technology has been shown to offer significant advantages, including low cost, high throughput, and broad target coverage. Consequently, this technology has the capacity to simultaneously monitor common respiratory pathogens, thereby meeting the routine needs for infectious disease prevention and control. Concurrently, its rapid detection capabilities provide critical evidence for early clinical diagnosis, making it worthy of inclusion in routine clinical practice and the conduct of cost-effectiveness studies.

This study also has certain limitations. First, the study is based on cross-sectional PCR data and describes statistical patterns of co-detection among pathogens, rather than biological interactions or causal relationships. A positive bacterial PCR result may reflect only upper respiratory tract colonization rather than active infection, particularly in oropharyngeal or nasopharyngeal swab specimens. Second, specimen types were heterogeneous, with oropharyngeal swabs predominating and nasopharyngeal swabs and bronchoalveolar lavage fluid being underrepresented. Despite covariate adjustment and stratified analyses, the small sample sizes of the latter two subgroups limited statistical power, so these subgroup findings should be interpreted cautiously. Third, comorbidity and vaccination data were unavailable, limiting evaluation of their effects on detected pathogens. The absence of viral load data precluded assessment of its impact on co-detection patterns. Fourth, systematic and quota sampling may have introduced selection bias, limiting sample representativeness. Given the low detection rate of *Bordetella pertussis*, any co-detection results associated with it should be interpreted with caution. Finally, no multiple-testing correction was performed for the 190 pairwise comparisons, so some findings may represent false positives and require validation in cohort studies.

## 5 Conclusion

The study revealed that RV, IAV, and SARS-CoV-2 had the highest detection rates among viruses, while *H. influenzae*, SPN, and GAS were the top detected bacteria. Differences in the pathogen profiles were observed across different age groups and disease severity levels. This finding indicates that interventions which take into account age and disease severity should be implemented in infectious disease prevention and control practices. Virus-virus pairs were predominantly negatively co-detected, whereas bacteria-bacteria pairs were predominantly positively co-detected. Virus-bacteria pairs show both patterns, highlighting the importance of multipathogen testing in clinical practice.

## Data Availability

The original contributions presented in the study are included in the article/Supplementary material, further inquiries can be directed to the corresponding authors.
