# Supplementary material for: Pathogen profiles and co-detection characteristics of acute respiratory infections based on active surveillance in a large district of southern China
Source: Front Public Health. 2026 Jul 10;14:1854385. doi: 10.3389/fpubh.2026.1854385 (PMC13395998; doi:10.3389/fpubh.2026.1854385)
Supplement: Supplementary file 1 [file Supplementary_file_1.docx]

Supplementary materials

# Supplementary Tables and Figures

- 1. **Supplementary Tables**

**Supplementary Table S1.** Target genes of the 20 respiratory pathogens detected by the multiplex PCR assay.

| Respiratory Tests | Reagents | FAM Tagging Project | Detection of Target Genes | VIC Marking Project | Detection of Target Genes |
| --- | --- | --- | --- | --- | --- |
| 5 Respiratory Tests | Pipe #1 | SARS-CoV-2 | ORF1ab，N gene | Internal Reference | RnaseP gene |
|  | Pipe #2 | IAV | M gene | IBV | NP gene |
|  | Pipe #3 | AdV | penton base protein III | RV | 5’noncoding region |
| 15 Respiratory Tests |  |  |  |  |  |
|  | Pipe #1 | RSV | M gene | PIV | HN gene |
|  | Pipe #2 | HBoV | NS gene | HMPV | fusion glycoprotein gene |
|  | Pipe #3 | HCoV-NL63 | N gene | HCoV-HKU1 | N gene |
|  | Pipe #4 | HCoV-229E | N gene | HCoV-OC43 | N gene |
|  | Pipe #5 | B. pertussis | ptxP gene | EV | polyprotein gene |
|  | Pipe #6 | MP | P1 adhesion protein | C. pneumoniae | outer membrane protein A gene |
|  | Pipe #7 | GAS | spy1258 gene | H. influenzae | siaT gene |
|  | Pipe #8 | SPN | lytA gene,SP2020 gene | Internal Reference | RnaseP gene |

**Supplementary Table 2A.** Pathogen detection rates among patients with acute respiratory infections in Bao’an district, Shenzhen, 2024-2025

|  | **Any virus detected** | | | **Any bacteria detected** | | | **Any pathogens detected** | | | **Two or more pathogens detected** | | |
| --- | --- | --- | --- | --- | --- | --- | --- | --- | --- | --- | --- | --- |
| **Groups** | **All**  **(N=6199)** | **Pneumonia (N=614)** | **Non  Pneumonia (N=5585)** | **All**  **(N=6199)** | **Pneumonia (N=614)** | **Non  Pneumonia (N=5585)** | **All**  **(N=6199)** | **Pneumonia (N=614)** | **Non  Pneumonia (N=5585)** | **All**  **(N=6199)** | **Pneumonia (N=614)** | **Non  Pneumonia (N=5585)** |
| **All(N=6199)** | 3011 (48.6) | 265 (43.2) | 2746 (49.2) | 1754 (28.3) | 161 (26.2) | 1593 (28.5) | 3876 (62.5) | 340 (55.4) | 3536 (63.3) | 1148 (18.5) | 106 (17.3) | 1042 (18.7) |
| **Sex** |  |  |  |  |  |  |  |  |  |  |  |  |
| Male(N=3353) | 1618 (48.3) | 148 (41.9) | 1470 (49.0) | 957 (28.5) | 90 (25.5) | 867 (28.9) | 2080 (62.0) | 189 (53.5) | 1891 (63.0) | 646 (19.3) | 65 (18.4) | 581 (19.4) |
| Female(N=2846) | 1393 (49.0) | 117 (44.8) | 1276 (49.4) | 797 (28.0) | 71 (27.2) | 726 (28.1) | 1796 (63.1) | 151 (57.8) | 1645 (63.6) | 502 (17.6) | 41 (15.7) | 461 (17.8) |
| χ2 | 0.267 | 0.403 | 0.059 | 0.193 | 0.146 | 0.413 | 0.710 | 0.962 | 0.192 | 2.596 | 0.591 | 2.050 |
| *P* | 0.605 | 0.525 | 0.808 | 0.660 | 0.702 | 0.520 | 0.399 | 0.327 | 0.661 | 0.107 | 0.442 | 0.152 |
| **Age group** |  |  |  |  |  |  |  |  |  |  |  |  |
| Children (<5 years) (N=1437) | 993 (69.1) | 175 (74.8) | 818 (68.0) | 539 (37.5) | 86 (36.8) | 453 (37.7) | 1162 (80.9) | 199 (85.0) | 963 (80.0) | 501 (34.9) | 77 (32.9) | 424 (35.2) |
| School age children  (5-17 years) (N=1022) | 557 (54.5) | 36 (53.7) | 521 (54.6) | 487 (47.6) | 33 (49.2) | 454 (47.5) | 793 (77.6) | 54 (80.6) | 739 (77.4) | 327 (32.0) | 18 (26.9) | 309 (32.4) |

**Supplementary Table 2B.** Pathogen detection rates among patients with acute respiratory infections in Bao’an district, Shenzhen, 2024-2025

|  | **Any virus detected** | | | **Any bacteria detected** | | | **Any pathogens detected** | | | **Two or more pathogens detected** | | |
| --- | --- | --- | --- | --- | --- | --- | --- | --- | --- | --- | --- | --- |
| **Groups** | **All**  **(N=6199)** | **Pneumonia (N=614)** | **Non  Pneumonia (N=5585)** | **All**  **(N=6199)** | **Pneumonia (N=614)** | **Non  Pneumonia (N=5585)** | **All**  **(N=6199)** | **Pneumonia (N=614)** | **Non  Pneumonia (N=5585)** | **All**  **(N=6199)** | **Pneumonia (N=614)** | **Non  Pneumonia (N=5585)** |
| **Age group** |  |  |  |  |  |  |  |  |  |  |  |  |
| Adult (18-59 years) (N=3465) | 1395 (40.3) | 36 (17.5) | 1359 (41.7) | 685 (19.8) | 26 (12.6) | 659 (20.2) | 1824 (52.6) | 58 (28.2) | 1766 (54.2) | 305 (8.8) | 5 (2.4) | 300 (9.2) |
| Old people (≥60 years） (N=275) | 66 (24.0) | 18 (16.8) | 48 (28.6) | 43 (15.6) | 16 (15.0) | 27 (16.1) | 97 (35.3) | 29 (27.1) | 68 (40.5) | 15 (5.5) | 6 (5.6) | 9 (5.4) |
| χ2 | 419.172 | 184.112 | 282.973 | 394.732 | 58.504 | 341.563 | 536.913 | 196.972 | 381.231 | 625.350 | 86.335 | 547.643 |
| *P* | ＜0.001 | ＜0.001 | ＜0.001 | ＜0.001 | ＜0.001 | ＜0.001 | ＜0.001 | ＜0.001 | ＜0.001 | ＜0.001 | ＜0.001 | ＜0.001 |
| Z | -20.466 | -12.988 | -16.821 | 0.899 | 58.504 | 0.516 | -7.749 | 7.024 | 6.454 | -9.592 | 5.471 | 8.539 |
| *P* | ＜0.001 | ＜0.001 | ＜0.001 | 0.3688 | ＜0.001 | 0.606 | ＜0.001 | ＜0.001 | ＜0.001 | ＜0.001 | ＜0.001 | ＜0.001 |
| **Case type** |  |  |  |  |  |  |  |  |  |  |  |  |
| Outpatients(N=5261) | 2545 (48.4) | 45 (34.4) | 2500 (48.7) | 1422 (27.0) | 17 (13.0) | 1405 (27.4) | 3272 (62.2) | 55 (42.0) | 3217 (62.7) | 910 (17.3) | 9 (6.9) | 901 (17.6) |

**Supplementary Table 2C.** Pathogen detection rates among patients with acute respiratory infections in Bao’an district, Shenzhen, 2024-2025

|  | **Any virus detected** | | | **Any bacteria detected** | | | **Any pathogens detected** | | | **Two or more pathogens detected** | | |
| --- | --- | --- | --- | --- | --- | --- | --- | --- | --- | --- | --- | --- |
| **Groups** | **All**  **(N=6199)** | **Pneumonia (N=614)** | **Non  Pneumonia (N=5585)** | **All**  **(N=6199)** | **Pneumonia (N=614)** | **Non  Pneumonia (N=5585)** | **All**  **(N=6199)** | **Pneumonia (N=614)** | **Non  Pneumonia (N=5585)** | **All**  **(N=6199)** | **Pneumonia (N=614)** | **Non  Pneumonia (N=5585)** |
| **Case type** |  |  |  |  |  |  |  |  |  |  |  |  |
| Inpatients(N=938) | 466 (49.7) | 220 (45.6) | 246 (54.1) | 332 (35.4) | 144 (29.8) | 188 (41.3) | 604 (64.4) | 285 (59.0) | 319 (70.1) | 238 (25.4) | 97 (20.1) | 141 (31.0) |
| χ2 | 0.492 | 4.820 | 4.545 | 27.047 | 14.242 | 39.102 | 1.550 | 11.403 | 9.538 | 33.876 | 11.687 | 48.757 |
| *P* | 0.483 | 0.028 | 0.033 | ＜0.001 | ＜0.001 | ＜0.001 | 0.2131 | ＜0.001 | 0.002 | ＜0.001 | ＜0.001 | ＜0.001 |

**Supplementary Table 3.** Detection rates of viral and bacterial pathogens among patients with acute respiratory infections in Bao’an district, Shenzhen, 2024-2025

| Pathogens | Positive  cases | Number of  tests conducted | Pathogen detection rates (%) |
| --- | --- | --- | --- |
| H. influenzae | 1086 | 6199 | 17.52 |
| RV | 840 | 6199 | 13.55 |
| IAV | 727 | 6199 | 11.73 |
| SPN | 663 | 6199 | 10.70 |
| SARS-CoV-2 | 518 | 6199 | 8.36 |
| RSV | 298 | 6199 | 4.81 |
| PIV | 239 | 6199 | 3.86 |
| GAS | 196 | 6199 | 3.16 |
| HMPV | 165 | 6199 | 2.66 |
| AdV | 140 | 6199 | 2.26 |
| EV | 108 | 6199 | 1.74 |
| HBoV | 74 | 6199 | 1.19 |
| MP | 72 | 6199 | 1.16 |
| IBV | 66 | 6199 | 1.06 |
| HCoV-NL63 | 43 | 6199 | 0.69 |
| HCoV-OC43 | 27 | 6199 | 0.44 |
| C. pneumoniae | 25 | 6199 | 0.40 |
| HCoV-HKU1 | 23 | 6199 | 0.37 |
| HCoV-229E | 21 | 6199 | 0.34 |
| B. pertussis | 2 | 6199 | 0.03 |

**Supplementary Table 4.** Detection rates of viral and bacterial pathogens among outpatients and inpatients with acute respiratory infections in Bao’an district, Shenzhen, 2024-2025

| Pathogens | Outpatients | | | Inpatients | | | χ² | *P* |
| --- | --- | --- | --- | --- | --- | --- | --- | --- |
|  | Positive  cases | Number of  tests conducted | Pathogen detection rates (%) | Positive  cases | Number of  tests conducted | Pathogen detection rates (%) |  |  |
| H. influenzae | 935 | 5261 | 17.77 | 151 | 938 | 16.10 | 1.12 | 0.289 |
| RV | 749 | 5261 | 14.24 | 91 | 938 | 9.70 | 11.30 | 0.001 |
| IAV | 612 | 5261 | 11.63 | 115 | 938 | 12.26 | 0.22 | 0.639 |
| SARS-CoV-2 | 487 | 5261 | 9.26 | 31 | 938 | 3.31 | 36.70 | <0.001 |
| SPN | 465 | 5261 | 8.84 | 198 | 938 | 21.11 | 83.55 | <0.001 |
| PIV | 201 | 5261 | 3.82 | 38 | 938 | 4.05 | 0.08 | 0.777 |
| GAS | 180 | 5261 | 3.42 | 16 | 938 | 1.71 | 5.70 | 0.017 |
| RSV | 165 | 5261 | 3.14 | 133 | 938 | 14.18 | 120.43 | <0.001 |
| HMPV | 142 | 5261 | 2.70 | 23 | 938 | 2.45 | 0.16 | 0.689 |
| AdV | 116 | 5261 | 2.20 | 24 | 938 | 2.56 | 0.33 | 0.566 |
| EV | 72 | 5261 | 1.37 | 36 | 938 | 3.84 | 14.53 | <0.001 |
| IBV | 63 | 5261 | 1.20 | 3 | 938 | 0.32 | 3.70 | 0.054 |
| HBoV | 54 | 5261 | 1.03 | 20 | 938 | 2.13 | 4.10 | 0.043 |
| MP | 53 | 5261 | 1.01 | 19 | 938 | 2.03 | 4.01 | 0.045 |
| HCoV-NL63 | 39 | 5261 | 0.74 | 4 | 938 | 0.43 | 0.52 | 0.471 |
| HCoV-OC43 | 22 | 5261 | 0.42 | 5 | 938 | 0.53 | 0.10 | 0.752 |
| HCoV-HKU1 | 21 | 5261 | 0.40 | 2 | 938 | 0.21 | 0.42 | 0.517 |
| HCoV-229E | 16 | 5261 | 0.30 | 5 | 938 | 0.53 | 0.63 | 0.427 |
| C. pneumoniae | 14 | 5261 | 0.27 | 11 | 938 | 1.17 | 6.30 | 0.012 |
| B. pertussis | 2 | 5261 | 0.04 | 0 | 938 | 0.00 | 0.37 | 0.543 |

**Supplementary Table 5A.** Detection rates of viral and bacterial pathogens by age group among patients with acute respiratory infections in Bao’an district, Shenzhen 2024-2025

| Pathogens | ＜5 years | | | 5-17 years | | | 18-59 years | | | ≥60 years | | | χ² | *P* |
| --- | --- | --- | --- | --- | --- | --- | --- | --- | --- | --- | --- | --- | --- | --- |
|  | Positive  cases | Number of  tests conducted | Pathogen detection rates (%) | Positive  cases | Number of  tests conducted | Pathogen detection rates(%) | Positive  cases | Number of  tests conducted | Pathogen detection rates (%) | Positive  cases | Number of  tests conducted | Pathogen detection rates (%) |  |  |
| RV | 336 | 1437 | 23.38 | 196 | 1022 | 19.18 | 297 | 3465 | 8.57 | 11 | 275 | 4.00 | 268.42 | <0.001 |
| SPN | 318 | 1437 | 22.13 | 197 | 1022 | 19.28 | 134 | 3465 | 3.87 | 14 | 275 | 5.09 | 412.35 | <0.001 |
| H. influenzae | 272 | 1437 | 18.93 | 296 | 1022 | 28.96 | 492 | 3465 | 14.20 | 26 | 275 | 9.45 | 152.73 | <0.001 |
| RSV | 253 | 1437 | 17.61 | 22 | 1022 | 2.15 | 20 | 3465 | 0.58 | 3 | 275 | 1.09 | 401.28 | <0.001 |
| PIV | 138 | 1437 | 9.60 | 34 | 1022 | 3.33 | 58 | 3465 | 1.67 | 9 | 275 | 3.27 | 130.56 | <0.001 |
| IAV | 92 | 1437 | 6.40 | 157 | 1022 | 15.36 | 460 | 3465 | 13.28 | 18 | 275 | 6.55 | 128.91 | <0.001 |
| AdV | 73 | 1437 | 5.08 | 42 | 1022 | 4.11 | 24 | 3465 | 0.69 | 1 | 275 | 0.36 | 77.24 | <0.001 |
| HMPV | 64 | 1437 | 4.45 | 35 | 1022 | 3.42 | 60 | 3465 | 1.73 | 6 | 275 | 2.18 | 32.19 | <0.001 |
| HBoV | 64 | 1437 | 4.45 | 7 | 1022 | 0.68 | 3 | 3465 | 0.09 | 0 | 275 | 0.00 | 86.37 | <0.001 |
| EV | 62 | 1437 | 4.31 | 22 | 1022 | 2.15 | 23 | 3465 | 0.66 | 1 | 275 | 0.36 | 52.81 | <0.001 |
| SARS-CoV-2 | 57 | 1437 | 3.97 | 59 | 1022 | 5.77 | 386 | 3465 | 11.14 | 16 | 275 | 5.82 | 112.47 | <0.001 |
| GAS | 35 | 1437 | 2.44 | 87 | 1022 | 8.51 | 72 | 3465 | 2.08 | 2 | 275 | 0.73 | 121.03 | <0.001 |
| MP | 19 | 1437 | 1.32 | 27 | 1022 | 2.64 | 24 | 3465 | 0.69 | 2 | 275 | 0.73 | 23.52 | <0.001 |
| HCoV-NL63 | 15 | 1437 | 1.04 | 7 | 1022 | 0.68 | 20 | 3465 | 0.58 | 1 | 275 | 0.36 | 3.21 | 0.360 |
| HCoV-OC43 | 10 | 1437 | 0.70 | 2 | 1022 | 0.20 | 14 | 3465 | 0.40 | 1 | 275 | 0.36 | 3.12 | 0.373 |

**Supplementary Table 5B.** Detection rates of viral and bacterial pathogens by age group among patients with acute respiratory infections in Bao’an district, Shenzhen 2024-2025

| Pathogens | ＜5 years | | | 5-17 years | | | 18-59 years | | | ≥60 years | | | χ² | *P* |
| --- | --- | --- | --- | --- | --- | --- | --- | --- | --- | --- | --- | --- | --- | --- |
|  | Positive  cases | Number of  tests conducted | Pathogen detection rates (%) | Positive  cases | Number of  tests conducted | Pathogen detection rates(%) | Positive  cases | Number of  tests conducted | Pathogen detection rates (%) | Positive  cases | Number of  tests conducted | Pathogen detection rates (%) |  |  |
| IBV | 6 | 1437 | 0.42 | 16 | 1022 | 1.57 | 43 | 3465 | 1.24 | 1 | 275 | 0.36 | 10.25 | 0.017 |
| HCoV-HKU1 | 4 | 1437 | 0.28 | 8 | 1022 | 0.78 | 10 | 3465 | 0.29 | 1 | 275 | 0.36 | 3.05 | 0.384 |
| HCoV-229E | 4 | 1437 | 0.28 | 3 | 1022 | 0.29 | 14 | 3465 | 0.40 | 0 | 275 | 0.00 | 1.23 | 0.746 |
| B. pertussis | 2 | 1437 | 0.14 | 0 | 1022 | 0.00 | 0 | 3465 | 0.00 | 0 | 275 | 0.00 | 2.01 | 0.570 |
| C. pneumoniae | 2 | 1437 | 0.14 | 14 | 1022 | 1.37 | 9 | 3465 | 0.26 | 0 | 275 | 0.00 | 22.48 | <0.001 |

**Supplementary Table 6.** Detection rates of viral and bacterial pathogens by pneumonia status among patients with acute respiratory infections in Bao’an district, Shenzhen, 2024-2025

| Pathogens | Pneumonia | | | Non-pneumonia | | | χ² | *P* |
| --- | --- | --- | --- | --- | --- | --- | --- | --- |
|  | Positive  cases | Number of  tests conducted | Pathogen detection rates (%) | Positive  cases | Number of  tests conducted | Pathogen detection rates (%) |  |  |
| RSV | 94 | 614 | 15.31 | 204 | 5585 | 3.65 | 142.32 | <0.001 |
| SPN | 90 | 614 | 14.66 | 573 | 5585 | 10.26 | 11.74 | 0.001 |
| H. influenzae | 68 | 614 | 11.07 | 1018 | 5585 | 18.23 | 18.05 | <0.001 |
| RV | 50 | 614 | 8.14 | 790 | 5585 | 14.15 | 16.60 | <0.001 |
| IAV | 32 | 614 | 5.21 | 695 | 5585 | 12.44 | 27.30 | <0.001 |
| SARS-CoV-2 | 28 | 614 | 4.56 | 490 | 5585 | 8.77 | 12.70 | <0.001 |
| HMPV | 21 | 614 | 3.42 | 144 | 5585 | 2.58 | 1.20 | 0.273 |
| EV | 20 | 614 | 3.26 | 88 | 5585 | 1.58 | 7.60 | 0.006 |
| PIV | 15 | 614 | 2.44 | 224 | 5585 | 4.01 | 3.70 | 0.054 |
| MP | 15 | 614 | 2.44 | 57 | 5585 | 1.02 | 7.70 | 0.006 |
| AdV | 11 | 614 | 1.79 | 129 | 5585 | 2.31 | 0.60 | 0.439 |
| HBoV | 11 | 614 | 1.79 | 63 | 5585 | 1.13 | 1.40 | 0.237 |
| C. pneumoniae | 8 | 614 | 1.30 | 17 | 5585 | 0.30 | 7.80 | 0.005 |
| HCoV-229E | 6 | 614 | 0.98 | 15 | 5585 | 0.27 | 5.30 | 0.021 |
| GAS | 5 | 614 | 0.81 | 191 | 5585 | 3.42 | 11.80 | 0.001 |
| HCoV-OC43 | 3 | 614 | 0.49 | 24 | 5585 | 0.43 | 0.04 | 0.841 |
| IBV | 2 | 614 | 0.33 | 64 | 5585 | 1.15 | 3.20 | 0.074 |
| HCoV-NL63 | 1 | 614 | 0.16 | 42 | 5585 | 0.75 | 3.10 | 0.079 |
| HCoV-HKU1 | 0 | 614 | 0.00 | 23 | 5585 | 0.41 | 2.50 | 0.114 |
| B. pertussis | 0 | 614 | 0.00 | 2 | 5585 | 0.04 | 0.22 | 0.638 |

**Supplementary Table 7A.** Detection rates of viral and bacterial pathogens by age and pneumonia status among patients with acute respiratory infections in Bao’an district, Shenzhen, 2024-2025

| Case type | Age group | Pathogens | Positive  cases | Number of  tests conducted | Pathogen detection rates (%) |
| --- | --- | --- | --- | --- | --- |
| Pneumonia | ＜5 years | RSV | 88 | 234 | 37.61 |
|  |  | SPN | 60 | 234 | 25.64 |
|  |  | H. influenzae | 39 | 234 | 16.67 |
|  |  | RV | 33 | 234 | 14.10 |
|  |  | EV | 16 | 234 | 6.84 |
|  |  | SARS-CoV-2 | 13 | 234 | 5.56 |
|  |  | IAV | 10 | 234 | 4.27 |
|  |  | PIV | 10 | 234 | 4.27 |
|  |  | HMPV | 9 | 234 | 3.85 |
|  |  | HBoV | 9 | 234 | 3.85 |
|  |  | AdV | 7 | 234 | 2.99 |
|  |  | HCoV-229E | 3 | 234 | 1.28 |
|  |  | HCoV-OC43 | 2 | 234 | 0.85 |
|  |  | HCoV-NL63 | 1 | 234 | 0.43 |
|  |  | MP | 1 | 234 | 0.43 |
|  |  | IBV | 0 | 234 | 0.00 |
|  |  | HCoV-HKU1 | 0 | 234 | 0.00 |
|  |  | GAS | 0 | 234 | 0.00 |
|  |  | B. pertussis | 0 | 234 | 0.00 |
|  |  | C. pneumoniae | 0 | 234 | 0.00 |
|  | 5-17 years | IAV | 15 | 67 | 22.39 |
|  |  | SPN | 15 | 67 | 22.39 |
|  |  | H. influenzae | 14 | 67 | 20.90 |
|  |  | MP | 7 | 67 | 10.45 |
|  |  | RV | 6 | 67 | 8.96 |
|  |  | RSV | 5 | 67 | 7.46 |
|  |  | AdV | 2 | 67 | 2.99 |
|  |  | PIV | 2 | 67 | 2.99 |
|  |  | HBoV | 2 | 67 | 2.99 |
|  |  | EV | 2 | 67 | 2.99 |
|  |  | GAS | 2 | 67 | 2.99 |
|  |  | SARS-CoV-2 | 1 | 67 | 1.49 |
|  |  | HMPV | 1 | 67 | 1.49 |
|  |  | HCoV-OC43 | 1 | 67 | 1.49 |
|  |  | C. pneumoniae | 1 | 67 | 1.49 |

**Supplementary Table 7B.** Detection rates of viral and bacterial pathogens by age and pneumonia status among patients with acute respiratory infections in Bao’an district, Shenzhen, 2024-2025

| Case type | Age group | Pathogens | Positive  cases | Number of  tests conducted | Pathogen detection rates (%) |
| --- | --- | --- | --- | --- | --- |
| Pneumonia | 5-17 years | IBV | 0 | 67 | 0.00 |
|  |  | HCoV-NL63 | 0 | 67 | 0.00 |
|  |  | HCoV-HKU1 | 0 | 67 | 0.00 |
|  |  | HCoV-229E | 0 | 67 | 0.00 |
|  |  | B. pertussis | 0 | 67 | 0.00 |
|  | 18-59 years | SARS-CoV-2 | 8 | 206 | 3.88 |
|  |  | HMPV | 8 | 206 | 3.88 |
|  |  | RV | 8 | 206 | 3.88 |
|  |  | SPN | 8 | 206 | 3.88 |
|  |  | C. pneumoniae | 7 | 206 | 3.40 |
|  |  | H. influenzae | 7 | 206 | 3.40 |
|  |  | MP | 6 | 206 | 2.91 |
|  |  | IAV | 4 | 206 | 1.94 |
|  |  | HCoV-229E | 3 | 206 | 1.46 |
|  |  | GAS | 3 | 206 | 1.46 |
|  |  | AdV | 2 | 206 | 0.97 |
|  |  | IBV | 1 | 206 | 0.49 |
|  |  | RSV | 1 | 206 | 0.49 |
|  |  | PIV | 1 | 206 | 0.49 |
|  |  | EV | 1 | 206 | 0.49 |
|  |  | HCoV-NL63 | 0 | 206 | 0.00 |
|  |  | HCoV-HKU1 | 0 | 206 | 0.00 |
|  |  | HCoV-OC43 | 0 | 206 | 0.00 |
|  |  | HBoV | 0 | 206 | 0.00 |
|  |  | B. pertussis | 0 | 206 | 0.00 |
|  | ≥60 years | H. influenzae | 8 | 107 | 7.48 |
|  |  | SPN | 7 | 107 | 6.54 |
|  |  | SARS-CoV-2 | 6 | 107 | 5.61 |
|  |  | IAV | 3 | 107 | 2.80 |
|  |  | HMPV | 3 | 107 | 2.80 |
|  |  | RV | 3 | 107 | 2.80 |
|  |  | PIV | 2 | 107 | 1.87 |
|  |  | IBV | 1 | 107 | 0.93 |
|  |  | EV | 1 | 107 | 0.93 |
|  |  | MP | 1 | 107 | 0.93 |

**Supplementary Table 7C.** Detection rates of viral and bacterial pathogens by age and pneumonia status among patients with acute respiratory infections in Bao’an district, Shenzhen, 2024-2025

| Case type | Age group | Pathogens | Positive  cases | Number of  tests conducted | Pathogen detection rates (%) |
| --- | --- | --- | --- | --- | --- |
| Pneumonia | ≥60 years | RSV | 0 | 107 | 0.00 |
|  |  | AdV | 0 | 107 | 0.00 |
|  |  | HCoV-NL63 | 0 | 107 | 0.00 |
|  |  | HCoV-HKU1 | 0 | 107 | 0.00 |
|  |  | HCoV-229E | 0 | 107 | 0.00 |
|  |  | HCoV-OC43 | 0 | 107 | 0.00 |
|  |  | HBoV | 0 | 107 | 0.00 |
|  |  | GAS | 0 | 107 | 0.00 |
|  |  | B. pertussis | 0 | 107 | 0.00 |
|  |  | C. pneumoniae | 0 | 107 | 0.00 |
| Non-Pneumonia | ＜5 years | RV | 303 | 1203 | 25.19 |
|  |  | SPN | 258 | 1203 | 21.45 |
|  |  | H. influenzae | 233 | 1203 | 19.37 |
|  |  | RSV | 165 | 1203 | 13.72 |
|  |  | PIV | 128 | 1203 | 10.64 |
|  |  | IAV | 82 | 1203 | 6.82 |
|  |  | AdV | 66 | 1203 | 5.49 |
|  |  | HMPV | 55 | 1203 | 4.57 |
|  |  | HBoV | 55 | 1203 | 4.57 |
|  |  | EV | 46 | 1203 | 3.82 |
|  |  | SARS-CoV-2 | 44 | 1203 | 3.66 |
|  |  | GAS | 35 | 1203 | 2.91 |
|  |  | MP | 18 | 1203 | 1.50 |
|  |  | HCoV-NL63 | 14 | 1203 | 1.16 |
|  |  | HCoV-OC43 | 8 | 1203 | 0.67 |
|  |  | IBV | 6 | 1203 | 0.50 |
|  |  | HCoV-HKU1 | 4 | 1203 | 0.33 |
|  |  | B. pertussis | 2 | 1203 | 0.17 |
|  |  | C. pneumoniae | 2 | 1203 | 0.17 |
|  |  | HCoV-229E | 1 | 1203 | 0.08 |
|  | 5-17 years | H. influenzae | 282 | 955 | 29.53 |
|  |  | RV | 190 | 955 | 19.90 |
|  |  | SPN | 182 | 955 | 19.06 |
|  |  | IAV | 142 | 955 | 14.87 |
|  |  | GAS | 85 | 955 | 8.90 |

**Supplementary Table 7D.** Detection rates of viral and bacterial pathogens by age and pneumonia status among patients with acute respiratory infections in Bao’an district, Shenzhen, 2024-2025

| Case type | Age group | Pathogens | Positive  cases | Number of  tests conducted | Pathogen detection rates (%) |
| --- | --- | --- | --- | --- | --- |
| Non-Pneumonia | 5-17 years | SARS-CoV-2 | 58 | 955 | 6.07 |
|  |  | AdV | 40 | 955 | 4.19 |
|  |  | HMPV | 34 | 955 | 3.56 |
|  |  | PIV | 32 | 955 | 3.35 |
|  |  | EV | 20 | 955 | 2.09 |
|  |  | MP | 20 | 955 | 2.09 |
|  |  | RSV | 17 | 955 | 1.78 |
|  |  | IBV | 16 | 955 | 1.68 |
|  |  | C. pneumoniae | 13 | 955 | 1.36 |
|  |  | HCoV-HKU1 | 8 | 955 | 0.84 |
|  |  | HCoV-NL63 | 7 | 955 | 0.73 |
|  |  | HBoV | 5 | 955 | 0.52 |
|  |  | HCoV-229E | 3 | 955 | 0.31 |
|  |  | HCoV-OC43 | 1 | 955 | 0.10 |
|  |  | B. pertussis | 0 | 955 | 0.00 |
|  | 18-59 years | H. influenzae | 485 | 3259 | 14.88 |
|  |  | IAV | 456 | 3259 | 13.99 |
|  |  | SARS-CoV-2 | 378 | 3259 | 11.60 |
|  |  | RV | 289 | 3259 | 8.87 |
|  |  | SPN | 126 | 3259 | 3.87 |
|  |  | GAS | 69 | 3259 | 2.12 |
|  |  | PIV | 57 | 3259 | 1.75 |
|  |  | HMPV | 52 | 3259 | 1.60 |
|  |  | IBV | 42 | 3259 | 1.29 |
|  |  | AdV | 22 | 3259 | 0.68 |
|  |  | EV | 22 | 3259 | 0.68 |
|  |  | HCoV-NL63 | 20 | 3259 | 0.61 |
|  |  | RSV | 19 | 3259 | 0.58 |
|  |  | MP | 18 | 3259 | 0.55 |
|  |  | HCoV-OC43 | 14 | 3259 | 0.43 |
|  |  | HCoV-229E | 11 | 3259 | 0.34 |
|  |  | HCoV-HKU1 | 10 | 3259 | 0.31 |
|  |  | HBoV | 3 | 3259 | 0.09 |
|  |  | C. pneumoniae | 2 | 3259 | 0.06 |
|  |  | B. pertussis | 0 | 3259 | 0.00 |

**Supplementary Table 7E.** Detection rates of viral and bacterial pathogens by age and pneumonia status among patients with acute respiratory infections in Bao’an district, Shenzhen, 2024-2025

| Case type | Age group | Pathogens | Positive  cases | Number of  tests conducted | Pathogen detection rates (%) |
| --- | --- | --- | --- | --- | --- |
| Non-Pneumonia | ≥60 years | H. influenzae | 18 | 168 | 10.71 |
|  |  | IAV | 15 | 168 | 8.93 |
|  |  | SARS-CoV-2 | 10 | 168 | 5.95 |
|  |  | RV | 8 | 168 | 4.76 |
|  |  | PIV | 7 | 168 | 4.17 |
|  |  | SPN | 7 | 168 | 4.17 |
|  |  | RSV | 3 | 168 | 1.79 |
|  |  | HMPV | 3 | 168 | 1.79 |
|  |  | GAS | 2 | 168 | 1.19 |
|  |  | AdV | 1 | 168 | 0.60 |
|  |  | HCoV-NL63 | 1 | 168 | 0.60 |
|  |  | HCoV-HKU1 | 1 | 168 | 0.60 |
|  |  | HCoV-OC43 | 1 | 168 | 0.60 |
|  |  | MP | 1 | 168 | 0.60 |
|  |  | IBV | 0 | 168 | 0.00 |
|  |  | HCoV-229E | 0 | 168 | 0.00 |
|  |  | HBoV | 0 | 168 | 0.00 |
|  |  | EV | 0 | 168 | 0.00 |
|  |  | B. pertussis | 0 | 168 | 0.00 |
|  |  | C. pneumoniae | 0 | 168 | 0.00 |

**Supplementary Table 8A.** Pairwise pathogen co-detection associations among patients with acute respiratory infections in Bao’an district, Shenzhen, 2024-2025 adjusting for multiple pathogens

| Pathogens | SARS-CoV-2 | IAV | IBV | RSV | AdV | HMPV | PIV |
| --- | --- | --- | --- | --- | --- | --- | --- |
| SARS-CoV-2 | - | 0 | 0.077 (0.010-0.580)#& | 0.266 (0.132-0.538)#& | 0.179 (0.042-0.757)#& | 0.253 (0.119-0.537)#& | 0.160 (0.064-0.402)#& |
| IAV | 0 | - | 0 | 0.063 (0.015-0.267)#& | 0.214 (0.085-0.537)#& | 0.084 (0.020-0.346)#& | 0.087 (0.027-0.277)#& |
| IBV | 0.075 (0.010-0.562)#& | 0 | - | 1.505 (0.431-5.256) | 0.648 (0.086-4.882) | 0 | 0 |
| RSV | 0.288 (0.143-0.582)#& | 0.076 (0.018-0.312)#& | 1.063 (0.320-3.528) | - | 0.272 (0.094-0.782) | 0.104 (0.031-0.344)#& | 0.174 (0.082-0.369)#& |
| AdV | 0.186 (0.043-0.799)#& | 0.202 (0.081-0.509)#& | 0.594 (0.077-4.588) | 0.287 (0.093-0.888) | - | 0.160 (0.022-1.180) | 0.640 (0.301-1.359) |
| HMPV | 0.247 (0.116-0.524)#& | 0.079 (0.019-0.325)#& | 0 | 0.145 (0.044-0.482)#& | 0.139 (0.019-1.028) | - | 0.117 (0.028-0.488)#& |
| PIV | 0.145 (0.058-0.367)#& | 0.083 (0.026-0.264)#& | 0 | 0.181 (0.084-0.392)#& | 0.590 (0.278-1.251) | 0.117 (0.028-0.494)#& | - |
| HCoV-NL63 | 0 | 0 | 0.866 (0.110-6.804) | 0.111 (0.014-0.880) | 0 | 0 | 0.373 (0.049-2.856) |
| HCoV-HKU1 | 0 | 0.317 (0.071-1.407) | 0 | 0 | 1.206 (0.154-9.470) | 0 | 0.823 (0.101-6.742) |
| HCoV-229E | 0.428 (0.054-3.382) | 0 | 0 | 1.353 (0.228-8.040) | 0 | 0 | 0 |
| HCoV-OC43 | 0 | 0 | 0 | 0.644 (0.055-7.483) | 0.931 (0.119-7.285) | 0 | 0.672 (0.086-5.268) |
| HBoV | 0 | 0.322 (0.076-1.362) | 0 | 0.737 (0.262-2.074) | 0.839 (0.292-2.410) | 0.875 (0.113-6.771) | 0.588 (0.205-1.685) |
| RV | 0.146 (0.080-0.266)#& | 0.170 (0.112-0.257)#& | 0 | 0.357 (0.216-0.590)#& | 0.769 (0.496-1.194) | 0.128 (0.055-0.297)#& | 0.329 (0.212-0.510)#& |
| EV | 0.330 (0.099-1.097) | 0.090 (0.012-0.668)#& | 0 | 0.239 (0.111-0.518)#& | 0.243 (0.033-1.798) | 0 | 0.309 (0.092-1.040) |
| H. influenzae | 0.777 (0.575-1.049) | 0.890 (0.707-1.121) | 0.619 (0.303-1.266) | 0.733 (0.500-1.076) | 1.103 (0.724-1.681) | 1.533 (1.026-2.290)* | 1.077 (0.766-1.514) |
| GAS | 0.223 (0.087-0.570)#& | 0.374 (0.210-0.664)#& | 0.296 (0.040-2.219) | 0.180 (0.042-0.778)# | 0.262 (0.063-1.086) | 0.760 (0.328-1.764) | 0.171 (0.042-0.706)#& |
| B. pertussis | 0 | 0 | 0 | 0 | 0 | 0 | 11.017 (0.425-285.567) |
| SPN | 1.004 (0.648-1.556) | 2.143 (1.624-2.826)*& | 3.587 (1.743-7.381)*& | 1.763 (1.234-2.518)*& | 1.294 (0.826-2.029) | 2.425 (1.575-3.734)*& | 1.153 (0.784-1.695) |
| MP | 0.267 (0.033-2.162) | 0.083 (0.011-0.605)#& | 0 | 0 | 0.904 (0.271-3.013) | 2.639 (0.746-9.329) | 0.398 (0.094-1.689) |
| C. pneumoniae | 0.941 (0.103-8.632) | 0.230 (0.029-1.826) | 0 | 0 | 1.074 (0.136-8.454) | 0.909 (0.106-7.796) | 0 |

**Supplementary Table 8B.** Pairwise pathogen co-detection associations among patients with acute respiratory infections in Bao’an district, Shenzhen, 2024-2025 adjusting for multiple pathogens

| Pathogens | HCoV-NL63 | HCoV-HKU1 | HCoV-229E | HCoV-OC43 | HBoV | RV | EV |
| --- | --- | --- | --- | --- | --- | --- | --- |
| SARS-CoV-2 | 0 | 0 | 0.348 (0.042-2.904) | 0 | 0 | 0.154 (0.085-0.270)#& | 0.355 (0.107-1.186) |
| IAV | 0 | 0.353 (0.079-1.570) | 0 | 0 | 0.306 (0.072-1.303) | 0.166 (0.110-0.251)#& | 0.086 (0.012-0.631)#& |
| IBV | 0.810 (0.103-6.343) | 0 | 0 | 0 | 0 | 0 | 0 |
| RSV | 0.104 (0.013-0.824) | 0 | 1.093 (0.197-6.051) | 0.904 (0.092-8.850) | 0.697 (0.245-1.982) | 0.254 (0.157-0.410)#& | 0.209 (0.096-0.454)#& |
| AdV | 0 | 1.322 (0.164-10.643) | 0 | 0.734 (0.092-5.854) | 0.841 (0.290-2.439) | 0.818 (0.531-1.261) | 0.255 (0.034-1.913) |
| HMPV | 0 | 0 | 0 | 0 | 0.741 (0.092-5.982) | 0.112 (0.048-0.259)#& | 0 |
| PIV | 0.287 (0.037-2.225) | 0.834 (0.104-6.705) | 0 | 0.564 (0.071-4.500) | 0.563 (0.195-1.624) | 0.325 (0.211-0.499)#& | 0.261 (0.076-0.893) |
| HCoV-NL63 | - | 0 | 0 | 0 | 3.188 (0.605-16.791) | 0.137 (0.018-1.018) | 0 |
| HCoV-HKU1 | 0 | - | 0 | 0 | 0 | 0.318 (0.071-1.428) | 0 |
| HCoV-229E | 0 | 0 | - | 0 | 15.045 (1.457-155.385)* | 1.038 (0.228-4.712) | 0 |
| HCoV-OC43 | 0 | 0 | 0 | - | 0 | 0 | 0 |
| HBoV | 2.233 (0.387-12.871) | 0 | 6.851 (0.679-69.095) | 0 | - | 1.596 (0.948-2.684) | 0 |
| RV | 0.123 (0.016-0.932) | 0.296 (0.065-1.341) | 1.021 (0.226-4.607) | 0 | 1.566 (0.916-2.677) | - | 1.219 (0.699-2.124) |
| EV | 0 | 0 | 0 | 0 | 0 | 1.201 (0.703-2.051) | - |
| H. influenzae | 1.213 (0.572-2.572) | 0.228 (0.030-1.729) | 0.913 (0.254-3.290) | 0.366 (0.085-1.586) | 0.537 (0.250-1.154) | 1.017 (0.831-1.243) | 1.619 (1.015-2.580)*& |
| GAS | 1.306 (0.286-5.969) | 0 | 0 | 0 | 1.014 (0.232-4.438) | 0.676 (0.442-1.033) | 0.477 (0.111-2.057) |
| B. pertussis | 0 | 0 | 0 | 0 | 0 | 0 | 62.578 (2.546-1538.086)*& |
| SPN | 0.460 (0.103-2.049) | 0.324 (0.041-2.542) | 1.953 (0.463-8.242) | 1.489 (0.470-4.724) | 0.418 (0.192-0.912)#& | 1.162 (0.914-1.478) | 0.530 (0.273-1.030) |
| MP | 0 | 0 | 0 | 1.900 (0.231-15.648) | 0.483 (0.062-3.744) | 0.330 (0.146-0.744)#& | 0 |
| C. pneumoniae | 0 | 0 | 0 | 9.191 (0.907-93.123) | 0 | 0.252 (0.056-1.125) | 0 |

**Supplementary Table 8C.** Pairwise pathogen co-detection associations among patients with acute respiratory infections in Bao’an district, Shenzhen, 2024-2025 adjusting for multiple pathogens

| Pathogens | H. influenzae | GAS | B. pertussis | SPN | MP | C. pneumoniae |
| --- | --- | --- | --- | --- | --- | --- |
| SARS-CoV-2 | 0.715 (0.536-0.953)# | 0.229 (0.090-0.581)#& | 0 | 1.061 (0.703-1.601) | 0.316 (0.041-2.459) | 0.946 (0.106-8.417) |
| IAV | 0.920 (0.732-1.156) | 0.369 (0.207-0.657)#& | 0 | 2.100 (1.609-2.741)*& | 0.071 (0.010-0.521)#& | 0.144 (0.017-1.197) |
| IBV | 0.643 (0.318-1.300) | 0.278 (0.037-2.073) | 0 | 3.110 (1.536-6.295)*& | 0 | 0 |
| RSV | 0.874 (0.618-1.235) | 0.178 (0.042-0.750) | 0 | 1.930 (1.395-2.671)*& | 0 | 0 |
| AdV | 1.060 (0.698-1.610) | 0.255 (0.062-1.054) | 0 | 1.299 (0.831-2.032) | 0.760 (0.224-2.581) | 0.997 (0.122-8.125) |
| HMPV | 1.413 (0.957-2.087) | 0.690 (0.300-1.584) | 0 | 2.278 (1.492-3.477)*& | 2.114 (0.592-7.549) | 0.965 (0.102-9.149) |
| PIV | 1.071 (0.767-1.496) | 0.175 (0.043-0.721)#& | 6.070 (0.167-221.040) | 1.249 (0.858-1.821) | 0.373 (0.088-1.581) | 0 |
| HCoV-NL63 | 1.238 (0.594-2.579) | 1.782 (0.397-7.994) | 0 | 0.366 (0.082-1.624) | 0 | 0 |
| HCoV-HKU1 | 0.226 (0.030-1.701) | 0 | 0 | 0.406 (0.053-3.116) | 0 | 0 |
| HCoV-229E | 0.845 (0.240-2.979) | 0 | 0 | 1.655 (0.430-6.373) | 0 | 0 |
| HCoV-OC43 | 0.410 (0.096-1.756) | 0 | 0 | 1.615 (0.515-5.070) | 1.297 (0.156-10.797) | 8.770 (0.808-95.205) |
| HBoV | 0.537 (0.253-1.142) | 0.877 (0.204-3.763) | 0 | 0.444 (0.206-0.955)#& | 0.489 (0.064-3.758) | 0 |
| RV | 1.022 (0.838-1.248) | 0.713 (0.467-1.088) | 0 | 1.184 (0.934-1.502) | 0.289 (0.126-0.660)#& | 0.248 (0.053-1.156) |
| EV | 1.676 (1.073-2.619)*& | 0.445 (0.105-1.885) | 29.465 (1.152-753.585)*& | 0.605 (0.319-1.146) | 0 | 0 |
| H. influenzae | - | 0.782 (0.537-1.140) | 12.261 (0.303-495.385) | 2.105 (1.734-2.555)*& | 0.563 (0.272-1.164) | 1.099 (0.396-3.054) |
| GAS | 0.823 (0.569-1.192) | - | 0 | 1.686 (1.130-2.513)*& | 0 | 0.654 (0.082-5.184) |
| B. pertussis | 3.592 (0.217-59.462) | 0 | - | 0 | 0 | 0 |
| SPN | 2.116 (1.742-2.569)*& | 1.742 (1.162-2.611)*& | 0 | - | 0.266 (0.081-0.871)#& | 2.652 (0.982-7.161) |
| MP | 0.602 (0.294-1.231) | 0 | 0 | 0.282 (0.087-0.921)#& | - | 0.914 (0.154-5.415) |
| C. pneumoniae | 1.209 (0.444-3.297) | 0.529 (0.066-4.255) | 0 | 2.691 (1.045-6.931)* | 1.848 (0.357-9.582) | - |

^*^positive co-detection associations with two-sided p<0.05; ^#^negative co-detection associations with p<0.05. Column names represented the dependent variables, and row names represented the independent variables. ^&^represented the pairwise co-detection associations between ‘X’ and ‘Y’, as both the dependent and independent variables were consistent.A statistically significant co-detection association was referred to as a “multipathogen-adjusted association.”

**Supplementary Table 9A.** Pairwise pathogen co-detection associations in oropharyngeal swab samples from patients with acute respiratory infections in Bao'an district, Shenzhen, 2024-2025, adjusted for multiple pathogens

| Pathogens | SARS-CoV-2 | IAV | IBV | RSV | AdV | HMPV | PIV |
| --- | --- | --- | --- | --- | --- | --- | --- |
| SARS-CoV-2 | - | 0 | 0.078 (0.010-0.585)#& | 0.350 (0.166-0.739)#& | 0.107 (0.014-0.798)#& | 0.277 (0.124-0.618)#& | 0.183 (0.073-0.463)#& |
| IAV | 0 | - | 0 | 0.036 (0.005-0.265)#& | 0.228 (0.090-0.576)#& | 0.096 (0.023-0.400)#& | 0.093 (0.029-0.297)#& |
| IBV | 0.075 (0.010-0.563)#& | 0 | - | 1.635 (0.461-5.798) | 0.730 (0.096-5.527) | 0 | 0 |
| RSV | 0.380 (0.179-0.805)#& | 0.048 (0.007-0.353)#& | 1.192 (0.360-3.945) | - | 0.418 (0.144-1.217) | 0.136 (0.041-0.457)#& | 0.270 (0.125-0.580)#& |
| AdV | 0.110 (0.015-0.831)#& | 0.218 (0.086-0.550)#& | 0.652 (0.084-5.052) | 0.423 (0.134-1.337) | - | 0.199 (0.027-1.482) | 0.597 (0.267-1.339) |
| HMPV | 0.259 (0.115-0.580)#& | 0.088 (0.021-0.364)#& | 0 | 0.176 (0.052-0.596)#& | 0.163 (0.022-1.216) | - | 0.131 (0.031-0.549)#& |
| PIV | 0.165 (0.065-0.419)#& | 0.092 (0.029-0.294)#& | 0 | 0.299 (0.136-0.659)#& | 0.557 (0.249-1.245) | 0.128 (0.030-0.545)#& | - |
| HCoV-NL63 | 0 | 0 | 0.918 (0.115-7.308) | 0.169 (0.020-1.402) | 0 | 0 | 0.513 (0.065-4.043) |
| HCoV-HKU1 | 0 | 0.189 (0.024-1.479) | 0 | 0 | 1.662 (0.205-13.496) | 0 | 1.225 (0.136-11.003) |
| HCoV-229E | 0.693 (0.084-5.715) | 0 | 0 | 1.141 (0.123-10.597) | 0 | 0 | 0 |
| HCoV-OC43 | 0 | 0 | 0 | 0 | 0.989 (0.125-7.828) | 0 | 0.699 (0.087-5.593) |
| HBoV | 0 | 0.201 (0.027-1.496) | 0 | 1.011 (0.258-3.968) | 1.020 (0.350-2.977) | 1.057 (0.130-8.590) | 0.367 (0.086-1.568) |
| RV | 0.145 (0.075-0.279)#& | 0.195 (0.127-0.299)#& | 0 | 0.331 (0.177-0.619)#& | 0.826 (0.518-1.316) | 0.159 (0.067-0.375)#& | 0.333 (0.208-0.533)#& |
| EV | 0.253 (0.059-1.084) | 0.093 (0.012-0.689)#& | 0 | 0.250 (0.090-0.697)#& | 0 | 0 | 0.399 (0.117-1.360) |
| H. influenzae | 0.765 (0.559-1.049) | 0.911 (0.718-1.157) | 0.579 (0.275-1.220) | 0.668 (0.420-1.064) | 1.005 (0.633-1.595) | 1.374 (0.881-2.143) | 1.194 (0.833-1.711) |
| GAS | 0.231 (0.090-0.596)#& | 0.362 (0.199-0.660)#& | 0.314 (0.042-2.349) | 0.266 (0.062-1.144) | 0.290 (0.070-1.205) | 0.815 (0.347-1.912) | 0.094 (0.013-0.685)#& |
| B. pertussis | 0 | 0 | 0 | 0 | 0 | 0 | 8.616 (0.354-209.758) |
| SPN | 1.040 (0.651-1.660) | 2.302 (1.717-3.087)*& | 2.946 (1.362-6.372)*& | 2.050 (1.337-3.144)*& | 1.293 (0.794-2.104) | 2.368 (1.471-3.810)*& | 0.995 (0.646-1.531) |
| MP | 0 | 0.094 (0.013-0.692)#& | 0 | 0 | 0.938 (0.279-3.157) | 1.843 (0.402-8.445) | 0.399 (0.093-1.709) |
| C. pneumoniae | 0 | 0.299 (0.036-2.481) | 0 | 0 | 1.195 (0.149-9.589) | 0 | 0 |

**Supplementary Table 9B.** Pairwise pathogen co-detection associations in oropharyngeal swab samples from patients with acute respiratory infections in Bao'an district, Shenzhen, 2024-2025, adjusted for multiple pathogens

| Pathogens | HCoV-NL63 | HCoV-HKU1 | HCoV-229E | HCoV-OC43 | HBoV | RV | EV |
| --- | --- | --- | --- | --- | --- | --- | --- |
| SARS-CoV-2 | 0 | 0 | 0.604 (0.071-5.160) | 0 | 0 | 0.159 (0.082-0.306)#& | 0.262 (0.061-1.126) |
| IAV | 0 | 0.215 (0.027-1.686) | 0 | 0 | 0.191 (0.025-1.440) | 0.188 (0.123-0.288)#& | 0.082 (0.011-0.607)#& |
| IBV | 0.944 (0.118-7.523) | 0 | 0 | 0 | 0 | 0 | 0 |
| RSV | 0.201 (0.025-1.645) | 0 | 0.782 (0.068-9.060) | 0 | 1.113 (0.277-4.475) | 0.269 (0.150-0.483)#& | 0.219 (0.079-0.604)#& |
| AdV | 0 | 2.078 (0.250-17.263) | 0 | 0.754 (0.092-6.176) | 0.974 (0.330-2.874) | 0.882 (0.558-1.394) | 0 |
| HMPV | 0 | 0 | 0 | 0 | 1.032 (0.127-8.388) | 0.135 (0.058-0.316)#& | 0 |
| PIV | 0.355 (0.044-2.852) | 1.380 (0.156-12.203) | 0 | 0.621 (0.075-5.121) | 0.334 (0.078-1.432) | 0.330 (0.208-0.524)#& | 0.370 (0.107-1.279) |
| HCoV-NL63 | - | 0 | 0 | 0 | 6.310 (0.955-41.706) | 0.178 (0.023-1.354) | 0 |
| HCoV-HKU1 | 0 | - | 0 | 0 | 0 | 0.260 (0.033-2.056) | 0 |
| HCoV-229E | 0 | 0 | - | 0 | 0 | 1.770 (0.352-8.908) | 0 |
| HCoV-OC43 | 0 | 0 | 0 | - | 0 | 0 | 0 |
| HBoV | 10.099 (1.335-76.412) | 0 | 0 | 0 | - | 1.771 (0.970-3.231) | 0 |
| RV | 0.130 (0.016-1.073) | 0.238 (0.029-1.947) | 1.846 (0.385-8.845) | 0 | 1.615 (0.864-3.020) | - | 0.829 (0.415-1.653) |
| EV | 0 | 0 | 0 | 0 | 0 | 0.806 (0.415-1.567) | - |
| H. influenzae | 1.477 (0.643-3.394) | 0.272 (0.035-2.113) | 1.269 (0.324-4.976) | 0.395 (0.084-1.846) | 0.637 (0.262-1.549) | 1.050 (0.842-1.310) | 1.612 (0.950-2.736) |
| GAS | 1.848 (0.385-8.882) | 0 | 0 | 0 | 0.603 (0.078-4.661) | 0.734 (0.472-1.140) | 0.585 (0.134-2.548) |
| B. pertussis | 0 | 0 | 0 | 0 | 0 | 0 | 42.679 (1.893-961.988)*& |
| SPN | 0.576 (0.125-2.660) | 0.637 (0.080-5.087) | 0.768 (0.086-6.889) | 1.228 (0.330-4.570) | 0.310 (0.107-0.894)#& | 1.198 (0.917-1.565) | 0.650 (0.311-1.359) |
| MP | 0 | 0 | 0 | 1.899 (0.223-16.173) | 0.564 (0.072-4.402) | 0.239 (0.092-0.619)#& | 0 |
| C. pneumoniae | 0 | 0 | 0 | 38.589 (2.829-526.424) | 0 | 0.334 (0.072-1.558) | 0 |

**Supplementary Table 9C.** Pairwise pathogen co-detection associations in oropharyngeal swab samples from patients with acute respiratory infections in Bao'an district, Shenzhen, 2024-2025, adjusted for multiple pathogens

| Pathogens | H. influenzae | GAS | B. pertussis | SPN | MP | C. pneumoniae |
| --- | --- | --- | --- | --- | --- | --- |
| SARS-CoV-2 | 0.708 (0.524-0.957)# | 0.245 (0.096-0.623)#& | 0 | 1.129 (0.729-1.749) | 0 | 0 |
| IAV | 0.926 (0.731-1.173) | 0.355 (0.195-0.646)#& | 0 | 2.181 (1.642-2.897)*& | 0.078 (0.010-0.573)#& | 0.183 (0.021-1.615) |
| IBV | 0.583 (0.279-1.221) | 0.305 (0.041-2.283) | 0 | 2.471 (1.148-5.320)*& | 0 | 0 |
| RSV | 0.826 (0.542-1.259) | 0.247 (0.058-1.051) | 0 | 2.077 (1.410-3.059)*& | 0 | 0 |
| AdV | 0.952 (0.601-1.508) | 0.282 (0.068-1.169) | 0 | 1.287 (0.793-2.089) | 0.757 (0.221-2.590) | 1.286 (0.155-10.676) |
| HMPV | 1.285 (0.834-1.979) | 0.766 (0.331-1.775) | 0 | 2.221 (1.391-3.548)*& | 1.343 (0.287-6.289) | 0 |
| PIV | 1.173 (0.823-1.672) | 0.098 (0.013-0.709)#& | 6.070 (0.167-221.040) | 1.082 (0.710-1.649) | 0.335 (0.078-1.439) | 0 |
| HCoV-NL63 | 1.445 (0.650-3.211) | 2.492 (0.539-11.516) | 0 | 0.439 (0.095-2.027) | 0 | 0 |
| HCoV-HKU1 | 0.298 (0.039-2.291) | 0 | 0 | 0.648 (0.081-5.217) | 0 | 0 |
| HCoV-229E | 1.229 (0.325-4.643) | 0 | 0 | 0.639 (0.075-5.441) | 0 | 0 |
| HCoV-OC43 | 0.507 (0.117-2.202) | 0 | 0 | 1.451 (0.394-5.345) | 1.645 (0.193-13.995) | 9.290 (0.983-97.243) |
| HBoV | 0.572 (0.239-1.372) | 0.479 (0.064-3.603) | 0 | 0.319 (0.111-0.921)#& | 0.565 (0.072-4.414) | 0 |
| RV | 1.051 (0.844-1.307) | 0.791 (0.512-1.222) | 0 | 1.199 (0.920-1.562) | 0.203 (0.077-0.533)#& | 0.368 (0.077-1.760) |
| EV | 1.623 (0.979-2.690)*& | 0.557 (0.130-2.383) | 29.465 (1.152-753.585)*& | 0.742 (0.367-1.150) | 0 | 0 |
| H. influenzae | - | 0.792 (0.536-1.170) | 12.261 (0.303-495.385) | 2.292 (1.856-2.830)*& | 0.469 (0.207-1.060) | 1.035 (0.322-3.327) |
| GAS | 0.830 (0.565-1.217) | - | 0 | 1.798 (1.189-2.720)*& | 0 | 0.530 (0.065-5.285) |
| B. pertussis | 3.713 (0.222-62.094) | 0 | - | 0 | 0 | 0 |
| SPN | 2.307 (1.867-2.851)*& | 1.901 (1.253-2.885)*& | 0 | - | 0.103 (0.014-0.754)#& | 2.860 (0.850-9.623) |
| MP | 0.516 (0.231-1.154) | 0 | 0 | 0.114 (0.016-0.836)#& | - | 0 |
| C. pneumoniae | 1.154 (0.371-3.587) | 0.541 (0.067-4.388) | 0 | 2.373 (0.760-7.406) | 0 | - |

^*^positive co-detection associations with two-sided p<0.05; ^#^negative co-detection associations with p<0.05. Column names represented the dependent variables, and row names represented the independent variables. ^&^represented the pairwise co-detection associations between ‘X’ and ‘Y’, as both the dependent and independent variables were consistent. A statistically significant co-detection associationwas referred to as a “multipathogen-adjusted association.”

**1.2 Supplementary Figures**

**

**

**Supplementary Figure 1.** Join-Point regression analysis of detection rates of predominant viruses by patient age. A red point indicates the mean positive rate of patients in terms of age. The colored segments indicate the fitting values of the Join-Point regression.Legends give the Annual Percent Change (APC) value of each fitted curve for each tested virus. *indicates that the APC is significantly different from zero at two-sided P < 0.05. The p values were not adjusted for multiple comparisons. The gray bars indicate the number of patients tested for each pathogen. (A) RV; (B) IAV; (C) SARS-CoV-2





**Supplementary Figure 2.** Join-Point regression analysis of detection rates of predominant bacterias by patient age. A red point indicates the mean positive rate of patients in terms of age. The colored segments indicate the fitting values of the Join-Point regression.Legends give the Annual Percent Change (APC) value of each fitted curve for each tested virus. *indicates that the APC is significantly different from zero at two-sided P < 0.05. The p values were not adjusted for multiple comparisons. The gray bars indicate the number of patients tested for each pathogen. (A) H. influenzae; (B) SPN; (C) GAS
